# Analysis of characteristic flavor compounds in coffee peels subjected to different thermal processing treatments based on GC–IMS and HS-SPME–GC–MS combined with chemical pattern recognition

**DOI:** 10.1016/j.fochx.2026.104256

**Published:** 2026-08-10

**Authors:** Congyan Meng, Jie Wu, Chunrong Yan, Jianqiang Wei, Yuanxing Zhang, Tianjun Yuan, Ying Hou

**Affiliations:** a College of Materials and Chemical Engineering, Southwest Forestry University, Kunming 650224, China; b Yuxi Academy of Inspection, Testing and Certification, Yuxi 653100, China; c Yunnan Vocational Institute of Energy Technology, Qujing 655001, China; d Agro-Products Processing Research Institute, Yunnan Academy of Agricultural Sciences, Kunming 650223, China

**Keywords:** Coffee peel, Microwave thermal processing treatment, Hot-air thermal processing treatment, Gas chromatography–ion mobility spectrometry (GC–IMS), Headspace solid-phase microextraction–gas chromatography–mass spectrometry (HS-SPME–GC–MS), Volatile compounds

## Abstract

This study investigated the effects of microwave thermal processing treatment (MD) and hot-air thermal processing treatment (HAD) on the volatile flavor compounds of coffee peel, using sun-dried (SG) samples as the control. GC–IMS and HS-SPME–GC–MS were combined with multivariate analyses (PCA and PLS-DA) and ROAV evaluation. A total of 62 and 56 volatile organic compounds (VOCs) were tentatively identified by GC–IMS and GC–MS, respectively, with ketones, aldehydes, alcohols, and esters representing the predominant classes. The two analytical methods exhibited complementary detection capabilities. PCA and PLS-DA clearly differentiated the three thermal drying treatments. Aroma-related compounds (VIP > 1 and ROAV ≥ 1) included 2-furanmethanethiol, 3-methylbutanal, (E)-2-decenal, ethyl heptanoate, hexanal, benzyl alcohol, linalool and β-ionone. Based on the nine core coffee flavor categories, SG exhibited a complex aroma profile characterized by herbal/vegetable, fruity, and nutty/cocoa notes; HAD was distinguished by highly pronounced floral notes; whereas MD presented both floral and roasted characteristics. The integrated analytical strategy effectively discriminated among different thermal processing treatments and identified putative aroma markers, which require further validation through olfactometry or sensory evaluation. This study provides a scientific basis for elucidating the flavor profile of coffee peel and supports the further development of coffee cascara tea products.

## Highlights

• For the first time, the GC–IMS and HS-SPME–GC–MS were employed to analyze the volatile organic compounds (VOCs) of coffee peels subjected to different drying processes.
• The results indicate that eight key differential compounds were identified through the integration of flavoromics and multivariate statistical analyses.
• This study provides a scientific basis for elucidating the flavor profile of coffee peels and supports the subsequent development of coffee cascara tea products.

## 1 Introduction

Coffee, a perennial evergreen crop belonging to the *Rubiaceae* family and the genus *Coffea* (Miao, 2023), is one of the world's most consumed beverages, alongside cocoa and tea (Myszkowska-Ryciak, 2025). In China, commercial coffee cultivation is mainly concentrated in two regions: Yunnan Province, where *Coffea arabica* L. (Arabica) predominates, and Hainan Province, where *Coffea canephora* L. (Robusta) is exclusively grown, among these, the former is the focus of this study. Coffee peel, a major by-product generated during coffee processing, accounts for a substantial proportion of the fresh fruit weight. It is estimated that coffee processing yields approximately 40%–45% waste relative to the raw coffee beans, with the peel alone contributing up to 29.9% (Bessada et al., 2018). Fresh coffee peel, characterized by high moisture content and delicate tissue, is highly susceptible to microbial contamination and spoilage (Aneta et al., 2014). Drying is an effective preservation method that not only prolongs shelf life but also enhances its added value. Previous studies have demonstrated that drying significantly reduces the water activity of fruits and vegetables, thereby inhibiting microbial growth and enzymatic activity and ultimately extending product stability (Mehmet et al., 2020). However, drying can also modify volatile flavor compounds, potentially affecting the sensory quality of the final product. Traditionally, sun-drying has been used to lower the moisture content of coffee peel to approximately 10%–12%. This process produces a product with reduced acidity, enhanced sweetness and increased body, although it often exhibits slightly lower clarity and flavor attributes reminiscent of ripe or fermented fruits. These characteristics are largely attributed to the mild drying conditions, which minimize the degradation of thermally labile compounds such as terpenes and alcohols while allowing enzymatic reactions to continue, thereby promoting the formation and retention of volatile metabolites. Although sun-drying effectively extends the shelf life of food, its relatively low processing temperature may limit the inactivation or transformation of certain heat-resistant compounds in fruit peels, potentially resulting in residual microorganisms or incomplete suppression of enzyme activity. Consequently, this may affect the product's storage stability and flavor consistency. At present, modern processing techniques, such as hot-air drying (Chen, Zhong, et al., 2024), vacuum freeze-drying (Liang et al., 2025) and microwave drying (Wang et al., 2025), have been widely applied to optimize food drying processes. Researchers including Sun et al. (2019), Tian et al. (2021), Li, Yang, et al. (2019), Huang et al. (2012) and Hu et al. (2018) have conducted comparative studies on various drying methods using materials such as *Toona sinensis*, scallions, coriander, ginger and tea. Their findings show that different drying treatments generally reduce the diversity and concentration of volatile compounds. Among these methods, vacuum freeze-drying, which operates under low-temperature and low-pressure conditions, provides the greatest retention of heat-sensitive flavor constituents and is therefore regarded as the most effective technique for preserving volatile substances.

Flavor compound analysis is a critical step in evaluating the quality of dried and processed coffee peel. Its flavor profile arises from the complex interactions among numerous volatile compounds and reactions such as the Maillard reaction and thermal degradation can significantly modify the types and concentrations of aromatic constituents (Dong et al., 2022). Hu et al. (2023) confirmed that aldehydes such as hexanal, heptanal, (E)-2-heptenal, decanal, phenylacetaldehyde, and cinnamaldehyde in coffee peels are primarily formed through intermediate or degradation products in the Maillard reaction, which impart fatty, fruity, floral, or spicy aromas to food, and 1-Octen-3-ol is a product of thermal degradation. With advances in food flavoromics, the integration of chemical analytical techniques (e.g., GC–IMS and GC–MS) with sensory evaluation has become an important and essential strategy for elucidating the relationship between chemical composition and perceived flavor (Chen, Ling, et al., 2024). As an emerging non-targeted analytical tool for volatile compounds, Gas chromatography–ion mobility spectrometry (GC–IMS) has been increasingly applied in food flavor research owing to its high sensitivity, rapid detection speed and intuitive data visualization (Xiang et al., 2025). After the processed sample enters the instrument with the carrier gas, it first undergoes initial separation through the gas chromatography column. The separated components then enter the ion mobility tube, where the analyte molecules are ionized in the ionization region. Under the influence of an electric field and a counter-flow drift gas, the ionized sample ions migrate and eventually reach the faraday plate for detection, achieving a second-stage separation (Liedtke et al., 2018; Mochalski et al., 2018). Gas chromatography–mass spectrometry (GC–MS), on the other hand, enables efficient separation and accurate quantification of volatile components (Liu et al., 2022). HS-SPME–GC–MS has been widely applied for comprehensive aroma profiling in fermented beverages and plant matrices (Herkenhoff et al., 2024a; Herkenhoff, Brödel, et al., 2026). GC–MS primarily detects volatile compounds with relatively large molecular weights and higher concentrations, typically with carbon chain lengths ranging from C8 to C20, whereas GC–IMS mainly identifies small, highly volatile compounds with lower concentrations, generally in the C4 to C10 range. The two techniques are therefore complementary and together expand the detectable range of volatile components. In particular, HS-SPME–GC–MS enables component identification by determining the relative molecular masses of volatile organic compounds and is considered a well-established technique for volatile analysis (Wu, Lin, et al., 2025). Although it offers excellent separation and identification performance, its effectiveness is somewhat limited when analyzing low-molecular weight and trace-level compounds (Li et al., 2023). In recent years, the combined application of GC–IMS and HS-SPME–GC–MS has been widely used in flavor studies of various fruits, vegetables and foods (Ge et al., 2020; Li et al., 2021; Qi et al., 2020). For example, Feng et al. (2022), Zhang et al. (2024), Zhao et al. (2025) and Huang (2025) employed this integrated approach to investigate volatile components of Sichuan pepper, fermented cheese, potato chips and tea leaves, respectively. This dual-technology strategy not only expands the detection range but also provides robust support for establishing a more comprehensive, multidimensional, and reliable flavor database. In particular, further validation through gas chromatography–olfactometry (GC–O), trained sensory panels, aroma recombination models, and omission experiments would facilitate the accurate identification of characteristic aroma-active compounds in coffee cascara. However, limited research has been conducted on applying this combined GC–IMS and HS-SPME–GC–MS technical system to examine the effects of different thermal processing treatment methods on the volatile composition of coffee peel.

This study uses sun-dried coffee peel as the raw material and combines GC–IMS and HS-SPME–GC–MS techniques to analyze the volatile components of coffee peel samples subjected to sun-drying (SG), microwave processing (MD), and hot-air processing (HAD). It systematically investigates the effects of different processing methods on their volatile flavor composition. Relative odor activity value (ROAV) analysis was employed to identify the putative aroma compounds (ROAV ≥1) in the three types of coffee peel. Chemometric methods, including principal component analysis (PCA) and partial least squares discriminant analysis (PLS-DA), were used for pattern recognition of flavor differences. Furthermore, putative aroma-related differential marker compounds among the three coffee peel samples were screened based on variable importance in projection (VIP) values. Integrated omics and flavoromic strategies have proven effective in linking metabolic pathways with aroma formation and sensory outcomes (Herkenhoff et al., 2023; Herkenhoff, Praia, et al., 2026).These approaches reveal the differences in aroma components between sun-dried and different thermal processing treatment samples and identify the aroma-active compounds contributing to the overall aroma profile of coffee peel. The results of this study aim to provide a theoretical basis for optimizing coffee peel processing techniques and for quality control in coffee peel tea products.

## 2 Materials and methods

### 2.1 Materials and instruments

Coffee peel (*Coffea arabica* L., Arabica cultivar) was collected from Baoshan, Yunnan Province, China. All samples were obtained from the same batch of coffee cherries at the fully ripe stage, with uniform fruit maturity. The external standard n-ketones (C4 - C9) for retention index calculation include the following reagents, n-Alkanones: 2-butanone, 2-pentanone, 2-hexanone, 2-heptanone, 2-octanone, and 2-nonanone, all of chromatographic grade, were purchased from Shanghai Aladdin Biochemical Technology Co., Ltd. 2-Ethylhexanol (chromatographic grade) was obtained from Tianjin Zhiyuan Chemical Reagent Co., Ltd.

FlavorSpec® flavor analyzer (G.A.S. GmbH, Germany); GCMS-TQ8050 NXnci SYSTEM gas chromatography–mass spectrometry system (Shimadzu Corporation, Japan); P80F20CN1PV-DGR (WO) microwave oven (Guangdong Galanz Microwave & Electrical Appliances Manufacturing Co., Ltd., China); XMTA-500T electric blast drying oven (Yuyao Keyang Instrument Co., Ltd., China); DFY-500 swing-type grinder (Wenling Linda Machinery Co., Ltd., China); 75 μm CAR/PDMS solid-phase microextraction fiber (Supelco, USA); 60-mesh standard sieve (Shaoxing Shangyu Daoxu Wusi Instrument Factory, China).

### 2.2 Sample preparation

Six batches of sun-dried coffee peel (*Coffea arabica* L. (Arabica)) were obtained from Yunnan Coffee Factory. The six batches are biological replicates (independent batches from the same plantation). Each batch was divided into three subsamples (SG, MD, HAD). Then each subsample was measured in three analytical replicates. From these, brightly colored and uniformly sized specimens were selected for two distinct processing treatments: microwave treatment (0.7 g, 300 W, 70 s) and oven treatment (0.5 g, 120 °C, 30 min). These parameters were determined based on preliminary response surface methodology (RSM) optimization, which identified conditions that maximized the extraction of chlorogenic acid, caffeine, and trigonelline from the coffee peel. Although the RSM optimization was originally designed for non-volatile precursors, these parameters were retained because preliminary trials indicated that they also produced distinct volatile profiles suitable for comparative flavor analysis. The chlorogenic acid, caffeine, and trigonelline in coffee are non-volatile precursor substances that determine the flavor and chemical characteristics of coffee. During the thermal process, they are closely related to the volatile components generated by pyrolysis reactions: chlorogenic acid hydrolyzes into acids and transforms into aromatic compounds, caffeine contributes to bitterness and mouthfeel, and trigonelline undergoes pyrolysis to produce pyrazines and other aromatic substances. Together, these three substances determine the final quality of the coffee (Makiso et al., 2024). All processed samples were stored at −4 °C prior to analysis. Before testing, the coffee peel samples were processed according to the respective treatments, ground into powder, and passed through a 60-mesh standard sieve for subsequent flavor quality assessment. The samples were designated as follows: SG for sun-dried samples, MD for microwave-processed samples, and HAD for hot-air oven-processed samples.

### 2.3 GC–IMS analysis

Following the method of Hou et al. (2023) with modifications. Accurately weigh 0.5 g of sample powder into a 20 mL standard headspace extraction vial, which was subsequently sealed, and incubated at 80 °C with a rotation speed of 500 r/min for 15 min. Then, 300 μL of the headspace gas was injected into the autosampler in splitless mode, with the injection needle temperature maintained at 85 °C. Each sample was analyzed in triplicate.

The GC–IMS instrumental conditions were set as follows: an MXT-WAX capillary column (30 m × 0.53 mm ID, 1.0 μm film thickness) was used and maintained at 60 °C. High-purity nitrogen (≥99.999%) served as the carrier gas, with the flow rate programmed as follows: initial flow of 2 mL/min held for 2 min, then increased linearly from 2 mL/min to 10 mL/min over 8 min (2–10 min), followed by a linear increase from 10 mL/min to 100 mL/min over 10 min (10–20 min), and finally held at 100 mL/min for 20 min (20–40 min). The IMS detector temperature was set at 45 °C, employing a tritium ionization source with nitrogen (>99.999%) as the drift gas, flowing at a constant rate of 75 mL/min through the drift tube. Qualitative analysis of target compounds was performed by retrieving and matching the retention indices of volatile compounds against the built-in NIST and IMS database within the instrument software. Furthermore, GC–IMS peak intensities were used only as relative signal responses for comparing SG, HAD, and MD samples, rather than as absolute concentrations. All samples were analyzed under identical experimental conditions, and peak intensities were normalized prior to statistical analysis to minimize analytical variability.

### 2.4 HS-SPME–GC–MS analysis

A 0.5 g aliquot of powdered sample was transferred into a 20 mL headspace vial, followed by the addition of 2 μL of a 10 μg/mL 2-ethylhexanol internal standard solution. The vial was then sealed and equilibrated at 65 °C for 10 min. Subsequently, a preconditioned solid-phase microextraction (SPME) fiber (CAR/PDMS, 75 μm; Supelco, USA), previously activated at 260 °C for 3 min, was exposed to the headspace for volatile extraction for 20 min. After extraction, the fiber was immediately inserted into the GC injection port and thermally desorbed at 260 °C for 5 min prior to chromatographic analysis.

GC–MS Conditions: the analysis was performed using an RTX-5MS capillary column (30 m × 0.25 mm × 0.25 μm) in splitless injection mode. The injector temperature was maintained at 260 °C. High-purity helium (99.999%) was used as the carrier gas at a constant flow rate of 1.00 mL/min. The gradient temperature program was set as follows: an initial temperature of 50 °C held for 2 min, ramped to 160 °C at 5 °C/min and held for 2 min, then increased to 260 °C at 10 °C/min and held for 1.5 min. MS conditions were an electron impact ionization energy of 70 eV, an ion source temperature of 230 °C, and an interface temperature of 280 °C. Data were acquired in full-scan mode over a mass range of *m*/*z* 35–350, with each sample analyzed in triplicate. Compounds were tentatively identified by comparing the mass spectra of chromatographic peaks with the NIST 17 library, retaining only those with a similarity index ≥80% (Jiang et al., 2021), which is determined to be a reliable detection result.

### 2.5 Calculation of relative content

The relative content of each measured component was calculated using the area normalization method (Wu, Chen, et al., 2025), as shown in Eq. (1):

(1) $$C_{i}=\frac{A_{i}}{A_{0}}\times 100$$

where *C*_*i*_ represents the relative content of the target component (%); *A*_*i*_ denotes the peak area of the individual component, and *A*_*0*_ is the total peak area of all volatile components.

### 2.6 Calculation of relative odor activity value (ROAV)

The ROAV of each volatile compound was calculated according to the method reported by Wu, Lin, et al. (2025). The calculation method is shown in Eq. (2):

(2) $${\mathrm{ROAV}}_{a}\left(\%\right)=\frac{C_{A}}{C_{\max}}\times \frac{T_{\max}}{T_{A}}\times 100$$

where *C*_*A*_ and *T*_*A*_ represent the relative content (%) or the peak area and odor threshold (mg/kg) of the target compound, respectively, and *C*_*max*_ and *T*_*max*_ denote the relative content (%) or the peak area and odor threshold (mg/kg) of the compound contributing most strongly to the sample's overall aroma (Song et al., 2025; Wang et al., 2026; Yan et al., 2024). The above formula is applicable for the calculation of GC–IMS and GC–MS data, and the GC–IMS and GC–MS data have already been normalized.

Where *C_i_* represents the peak area of the target volatile compound, and *C_max_* represents that of the compound with the highest odor activity value; *T_i_* is the odor threshold in air and *T_max_* is the largest odor threshold in air.

### 2.7 Statistical analysis

Univariate analysis of variance (ANOVA) was performed using SPSS 27.0 software. Experimental data are presented as the mean ± standard deviation (SD, *n* = 3 samples). Differences between groups were considered statistically significant at *P* < 0.05. Percentage stacked bar charts, Venn diagrams, radar charts, and PCA score plots were generated using Origin 2024. Multivariate data analysis was conducted in SIMCA (version 14.1), where a PLS-DA model was constructed and corresponding plots were generated. Metabolites with a variable importance in projection (VIP) value >1 were selected as potential marker compounds. The R^2^ and Q^2^ values are also applied to assess the performance of the PLS-DA model (Szymańska et al., 2012). A clustering heatmap was generated using the online platform https://www.chiplot.online.

## 3 Results and discussion

### 3.1 GC–IMS analysis

**Fig. 1:**
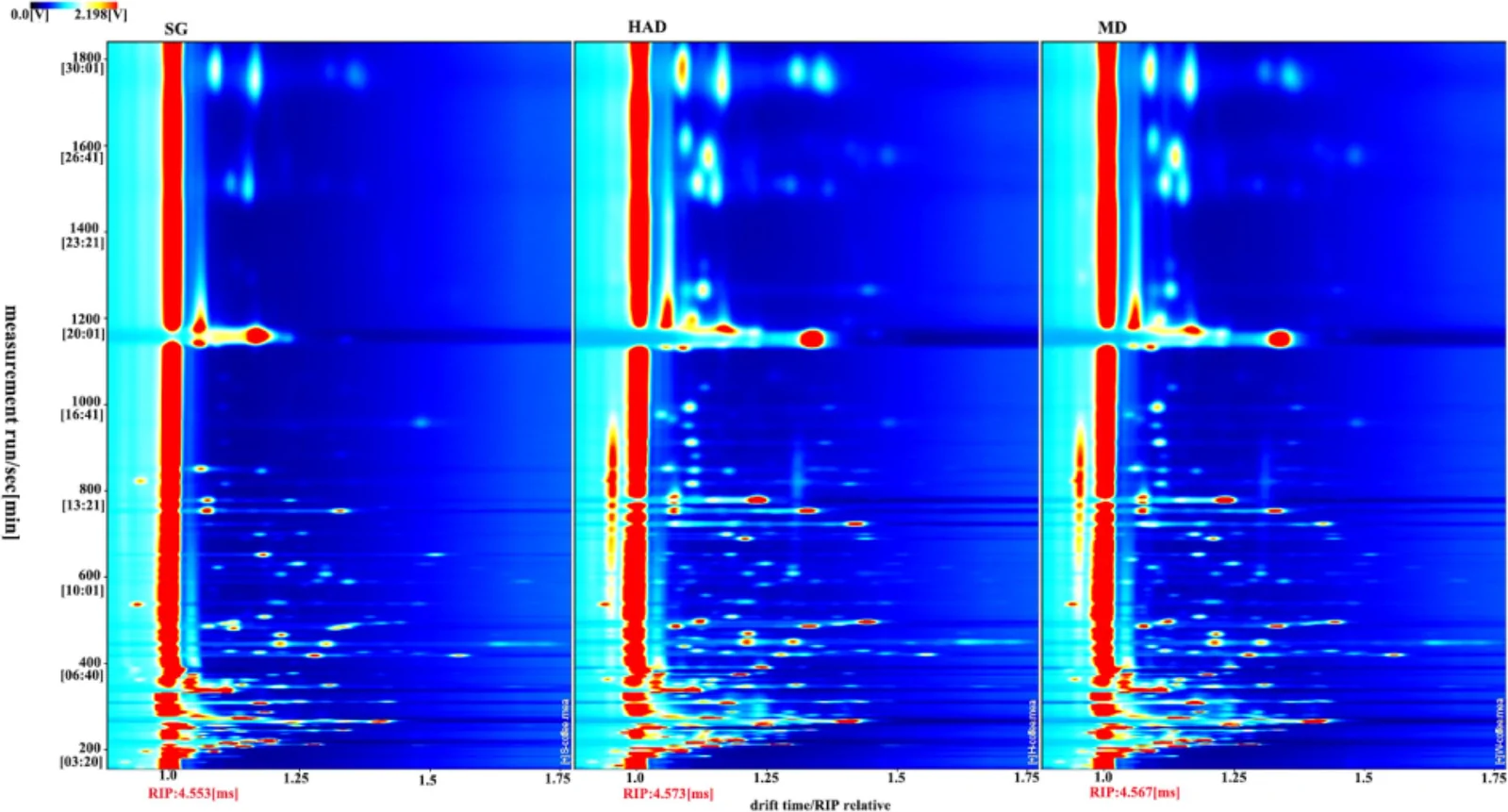
GC–IMS two-dimensional topographic plots of volatile compounds in SG, HAD and MD. The x-axis represents the normalized drift time (ms), the y-axis represents the retention time (s), and the color scale indicates the normalized signal intensity relative to the reaction ion peak (RIP). Higher signal intensities are represented by warmer colors (red), whereas lower signal intensities are represented by cooler colors (blue). Abbreviations: SG, sun-dried coffee peel; HAD, hot-air-dried coffee peel; MD, microwave-dried coffee peel. (For interpretation of the references to color in this figure legend, the reader is referred to the web version of this article.)

The flavor profiles of coffee peel processed using three different thermal processing methods were analyzed by GC–IMS, and the results are shown in Fig. 1. This two-dimensional topographic map was generated by projecting the three-dimensional spectral data (Yue et al., 2025). The red peak at the center of the figure is the Reaction Ion Peak (RIP), while each signal point to the right of the RIP corresponds to a volatile organic compound (VOC). The color intensity indicates the relative concentration of each compound, with red representing higher levels and white indicating lower levels. Overall, the abundance of volatile components differed among the different thermal processing treatment methods: the MD and HAD groups displayed generally higher concentrations, whereas the SG group exhibited comparatively lower levels and showed variations in specific compounds. These differences may be attributed to the thermal effects of microwave and hot-air oven drying, which can disrupt cell walls and promote the release of volatile constituents during continuous heating. When the concentration of the analyte is high, two or more molecules may share a proton or electron, forming dimers or even multimers. These aggregated ions exhibit retention times similar to those of their monomers but differ in drift time (Li, Li, et al., 2019). Within the retention time range of 200–1400 s and drift time 10 ms–15 ms, the volatile components of the three samples exhibited distinct clustered distribution characteristics, with some signal peaks extending even to retention times as high as 1800 s. This phenomenon may be attributed to the low polarity of certain compounds, resulting in stronger retention on the non-polar chromatographic column (Chen et al., 2018). Qualitative identification of the compounds was conducted using the instrument's built-in retention index database and the GC–IMS spectral library. A total of 68 volatile compounds were tentatively identified (detailed results are provided in Supplementary Table 1). The suffixes “M” and “D” following the compound names denote monomer and dimer, respectively. The identified compounds included 15 ketones, 14 aldehydes, 13 alcohols, 12 esters, 5 hydrocarbons, 4 ethers, 2 pyrazines and 1 each of furans, acids, and heterocyclic compounds.

**Fig. 2:**
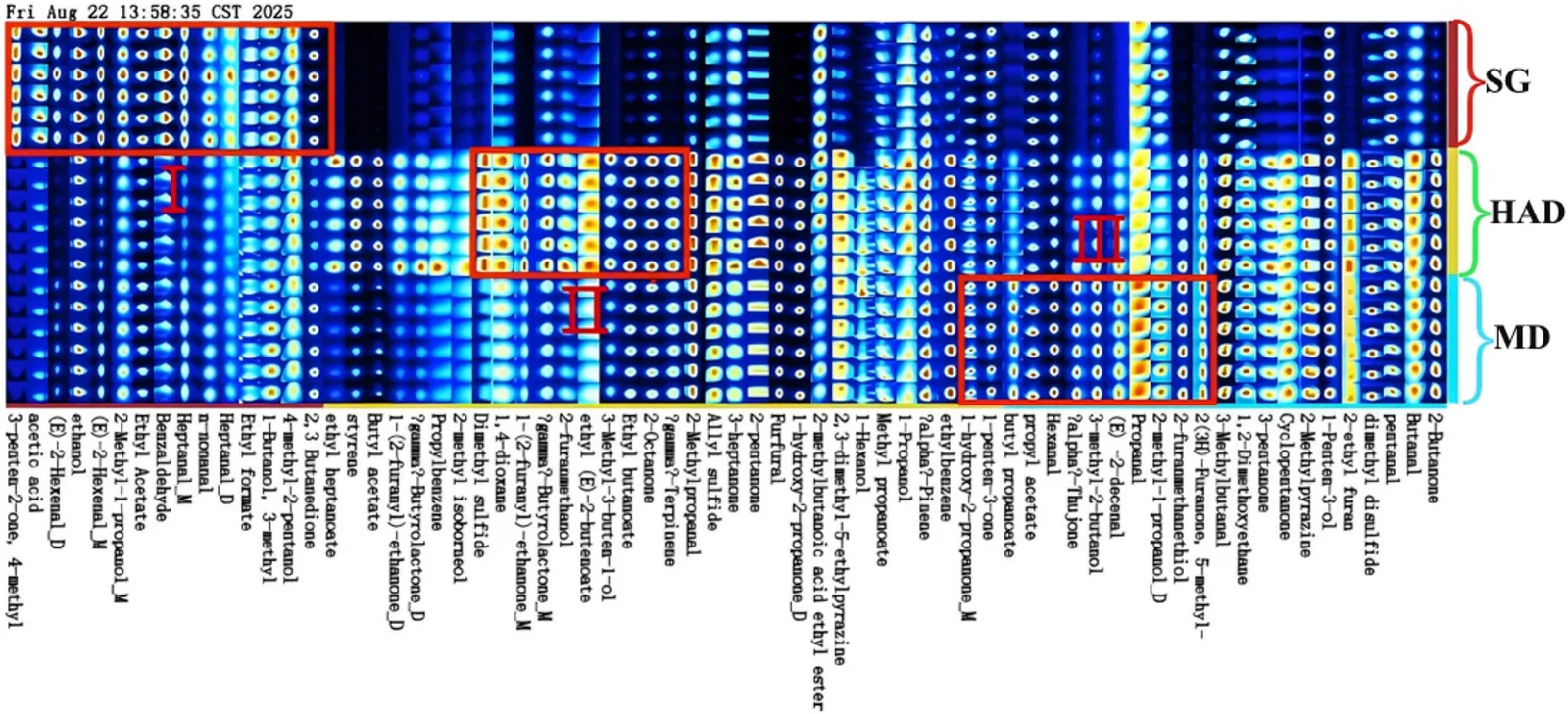
GC–IMS gallery plot fingerprints of volatile compounds in SG, HAD and MD. Each row represents an individual sample, and each column represents a volatile signal feature detected by GC–IMS. The color intensity indicates the normalized signal response, with darker colors representing higher signal intensities and lighter colors representing lower signal intensities.

For intuitively and quantitatively analyze the VOCs in coffee peel, the Gallery Plot plugin integrated within the GC–IMS system was employed to generate a fingerprint spectrum of volatile components (Fig. 2). This spectrum presents comprehensive VOC information for each sample, with each row representing a sample and each column corresponding to the signal distribution of a specific volatile compound. The varying color intensities reflect the relative abundance of each compound, with darker shades indicating higher concentrations. By comparing the fingerprint spectra of different samples, differences in VOC content between coffee peel subjected to various processing methods and the control group can be clearly distinguished. As illustrated in Fig. 2, the distribution of volatile substances can be broadly divided into three characteristic regions (I-III), corresponding to the signature volatile components of SG, HAD and MD samples, respectively. This classification highlights the differences in volatile compound profiles among the different processing methods. In region I, the signal intensity of volatile flavor compounds in the SG samples was markedly higher than those in the other processing groups. These elevated compounds include 4-methyl-3-penten-2-one, acetic acid, (E)-2-hexenal(D), ethanol, (E)-2-hexenal(M), 2-methylpropanol(M), ethyl acetate, benzaldehyde, heptanal(M), nonanal, heptanal(D), ethyl formate, 3-methylbutanol, 4-methyl-2-pentanol and 2,3-butanedione. Collectively, these compounds impart a complex blend of pungent, earthy, grassy, aromatic, sweet, fruity, fatty and floral notes to the SG samples. As exposure to sunlight increases, microbial fermentation, enzymatic reactions and oxidative processes occur within the peel, further promoting the formation of wine-like aromas and lipid compounds. In region II, the VOCs content of the HAD samples were significantly higher than that of other groups, with representative components including dimethyl sulfide, 1,4-dioxane, 1-(2-furanyl)-ethanone(M), γ-butyrolactone(M), 2-furanmethanol, ethyl(E)-2-butenoate, 3-methyl-3-buten-1-ol, ethyl butanoate, 2-octanone and γ-terpinene. These compounds contribute to the characteristic vegetable, smoky, buttery, roasted nut, toasted, rum and ether-like aromas, pineapple-like fruitiness, blue cheese mouldiness and pine wood notes observed in HAD samples. Region III exhibits higher VOCs contents in MD samples, predominantly comprising 1-hydroxy-2-propanone(M), 1-penten-3-one, butyl propanoate, propyl acetate, hexanal, *α*-thujone, 3-methyl-2-butanol, (E)-2-decenal, propanal, 2-methyl-1-propanol(D), 2-furanmethanethiol and 5-methyl-2(3H)-furanone. These compounds impart complex aromas to the MD samples, including caramel, nuts, sweet rose, cooling herbs, fruit, waxiness, coriander, green mushroom, aldehydic, grassiness and tobacco notes.

**Fig. 3:**
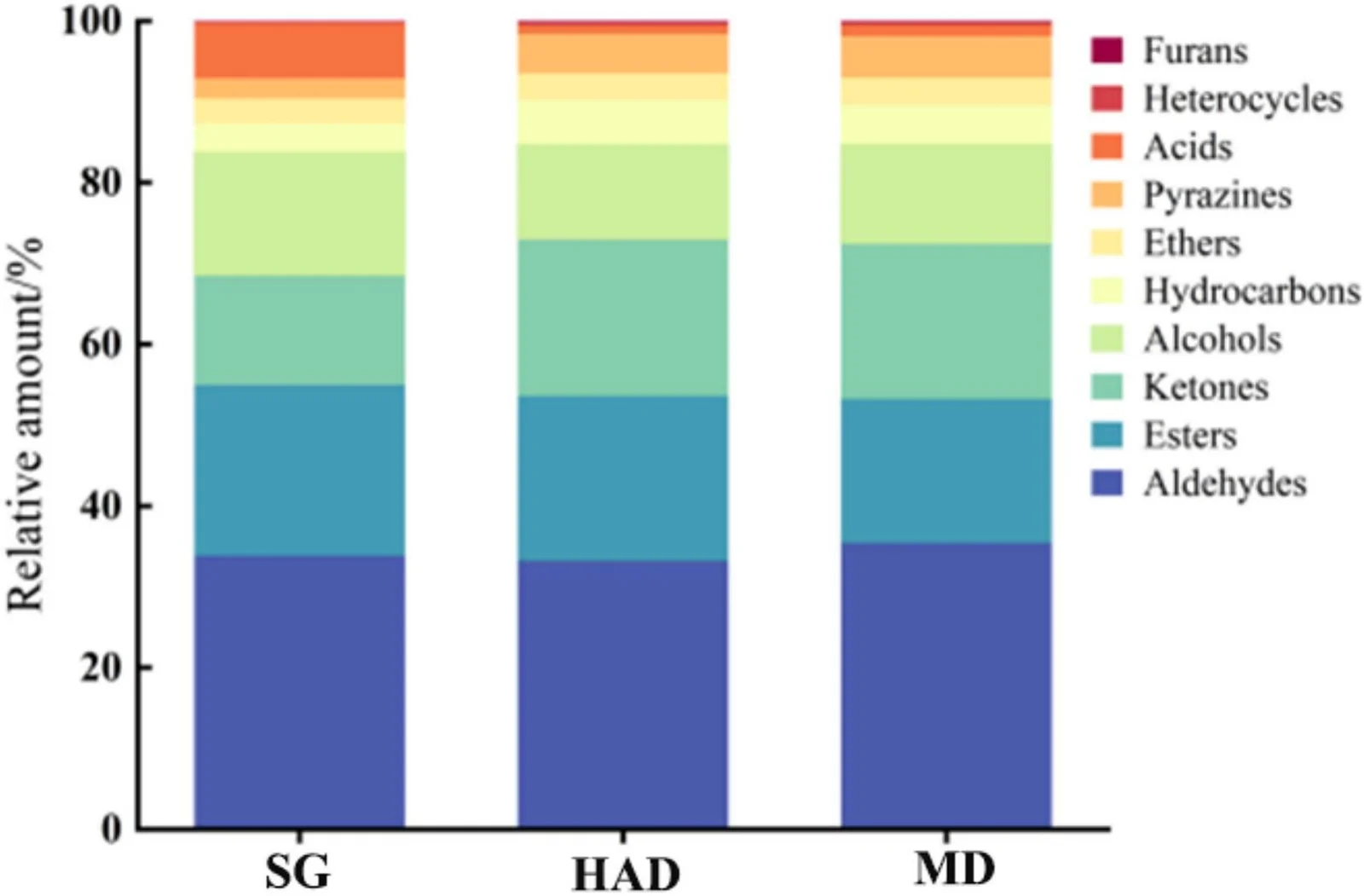
Relative proportions of the major chemical classes identified by GC–IMS in SG, HAD, and MD. The y-axis represents the relative percentage of the total normalized GC–IMS signal intensity attributed to each chemical class.

**Fig. 4:**
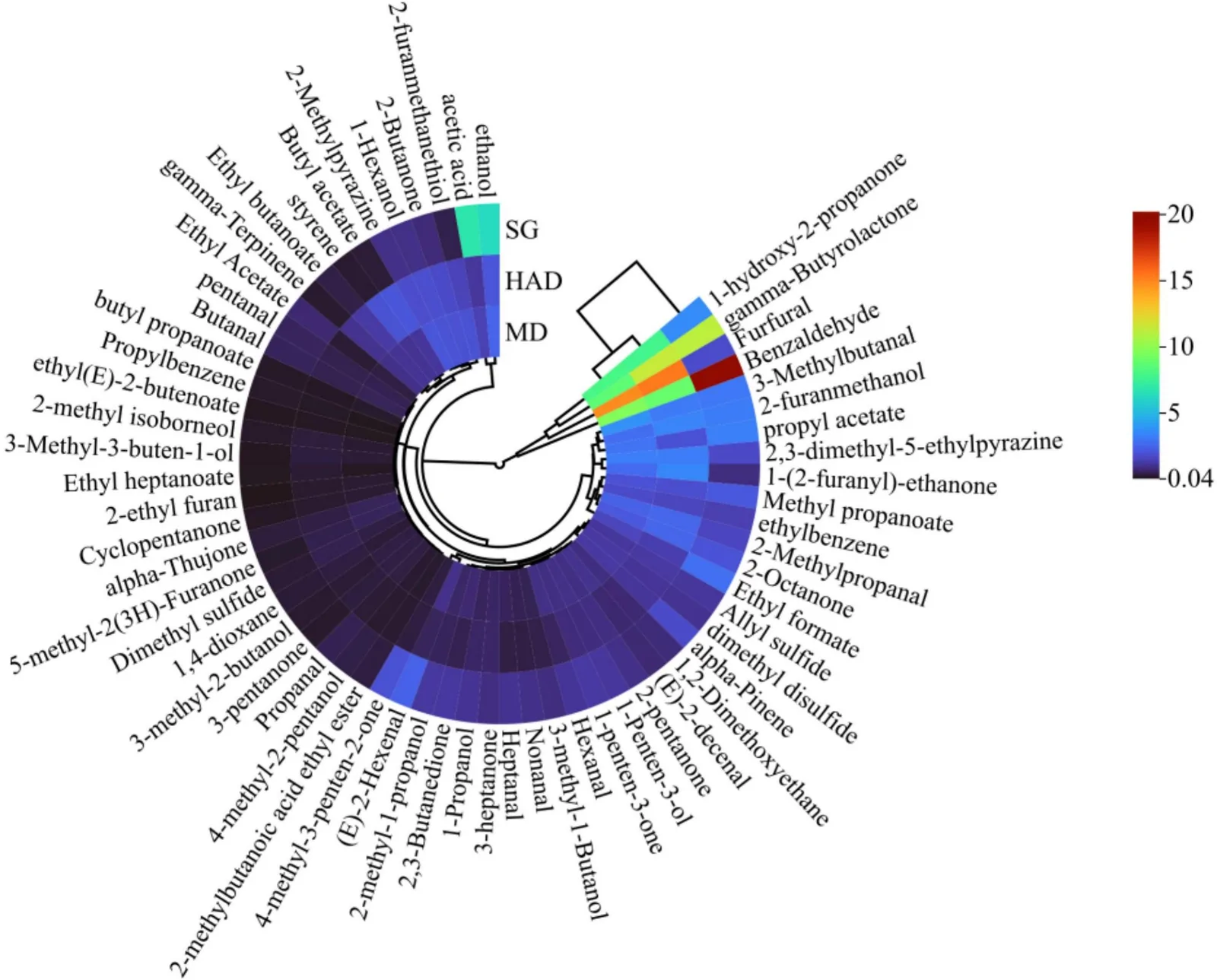
Hierarchical clustering heatmap of normalized GC–IMS signal responses in SG, HAD, and MD. Color intensity indicates the relative abundance of each volatile-ion feature among the different sample groups.

To systematically analyze the compositional characteristics of VOCs in coffee peel processed using three distinct thermal processing treatment methods, the categories and relative abundances of VOCs were visually characterized through relative proportion charts and cluster analysis heatmaps (Fig. 3 and Fig. 4). The results indicated that ketones, aldehydes, alcohols and esters were the predominant volatile components across all three types of coffee peel, collectively accounting for more than 84% of the total VOC content. Aldehydes, often associated with green and fresh aroma notes (Hou et al., 2023), were the most predominant volatile components in all three samples. Their relative contents were 33.835% in SG, 33.233% in HAD, and 35.423% in MD, with no statistically significant differences observed among them. This indicates that aldehyde levels remain relatively stable across the various thermal processing treatment processes. Following thermal processing treatment, the relative content of ketone increased (SG: 13.579%, HAD: 19.398%, MD: 19.252%), whereas the relative contents of alcohols (SG: 15.206%, HAD: 11.710%, MD: 12.324%) and esters (SG: 21.076%, HAD: 20.396%, MD: 17.838%) showed slight decreases. The relative content of acids declined markedly by up to 6%, likely due to the oxidation of fatty acids during processing. For instance, hexanal and 2-nonenal are commonly recognised as oxidation products of linoleic acid (Zhang et al., 2025). The cluster heatmap results showed that the volatile organic compounds (VOCs) of the three samples were grouped into distinct clusters, with MD and HAD falling into the same cluster, indicating that the types and contents of compounds in the MD and HAD samples were more similar. The relative contents of furfural, 1-hydroxy-2-propanone, 1-hexanol, 2-furfuryl mercaptan, 1-(2-furanyl)ethanone, 2-butanone, butyl acetate, ethyl butyrate, 2-methylpropanal, styrene, γ-terpinene, 2-methylpyrazine, and 2,3-dimethyl-5-ethylpyrazine significantly increased. The accumulation of these compounds imparted a complex flavor profile to the coffee peels, characterized by sweet, caramel, nutty, roasted, coffee, fatty, fruity, and woody notes. In contrast, the relative contents of benzaldehyde, acetic acid, ethanol, 2,3-butanedione, 4-methyl-3-penten-2-one, propyl acetate, ethyl formate, (E)-2-hexenal, and α-pinene markedly decreased after processing, leading to a pronounced attenuation of the originally prominent bitter almond, sharp acidic, irritating pungent, earthy, grassy, and piney odors in the peels. Overall, the cluster heatmap clearly revealed that the thermal processing treatment process promoted the formation of characteristic roasted and sweet aromatic compounds while inhibiting green and irritating off-odors, thereby optimizing the flavor quality of coffee peels. Benzaldehyde, formed through Strecker degradation or lipid oxidation, is reported to enhance almond, nutty, caramel and fruity aromas (Zhao et al., 2023). Additionally, heat treatment increased the levels of furan compounds, which contribute caramel, sweet, nutty and burnt aromas (Lotfy et al., 2021).

### 3.2 HS-SPME-GC–MS analysis

The volatile profiles of coffee peel processed by three different thermal processing methods were analyzed using HS-SPME-GC–MS. A total of 56 compounds were identified and categorized into 10 chemical categories (detailed results are provided in Supplementary Table 2). Among these, ketones (14 types) were the most abundant, followed by esters (11 types), aldehydes (9 types) and alcohols (8 types). Additional compounds included acids (4 types), alkenes (4 types), phenols (3 types) and single types of ethers, pyrroles and other compounds. Specifically, 37, 44 and 50 volatile flavor compounds were detected in the SG, HAD and MD samples, respectively, indicating that microwave processing (MD) promotes greater diversity in volatile composition. The study further revealed that different thermal processing treatment processes not only induce the formation of new volatile compounds in coffee peel but also result in the loss of certain original components, highlighting the significant modulatory effect of thermal processing treatment on the composition of volatile constituents.

**Fig. 5:**
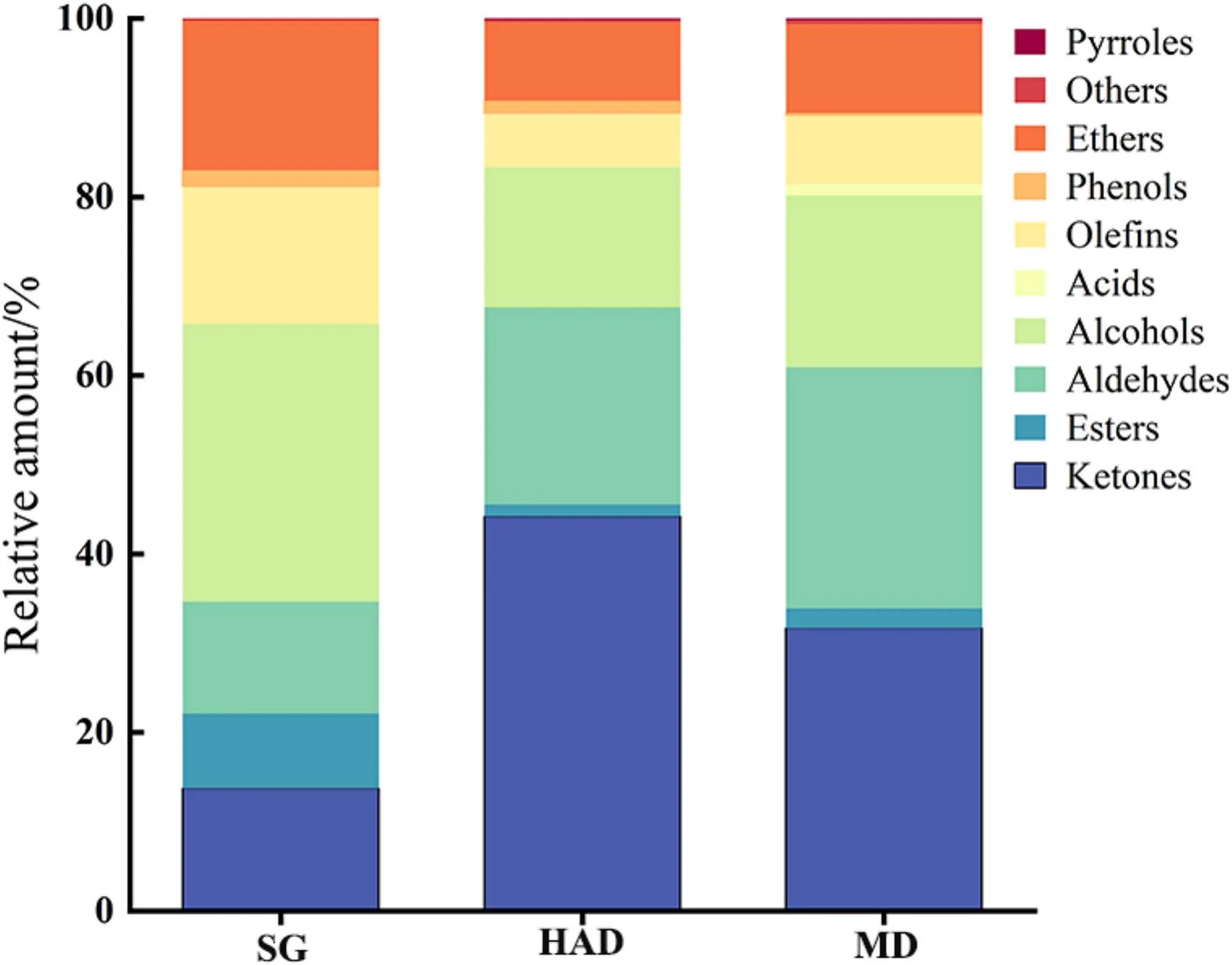
Relative proportions of major VOC classes based on GC–MS peak-area normalization in SG, HAD, and MD. The y-axis represents the percentage contribution of each VOC class to the total integrated GC–MS peak area.

Fig. 5 presents a stacked bar chart illustrating the differences in VOC contents among three coffee peel samples based on the similarity of their VOC compositional profiles. The results show that the predominant volatile components in the SG sample were ketones, esters, aldehydes, alcohols, alkenes and ethers, together accounting for 97.973% of the total volatile content. In the coffee peel samples subjected to different processing methods, the main volatile constituents were ketones, aldehydes, alcohols, alkenes and ethers, with their combined proportions reaching 96.845% in the HAD group and 95.715% in the MD group. Among the volatile compounds in the SG sample, alcohols exhibited the highest relative abundance (31.119%). The principal alcohols identified were benzyl alcohol (11.790%), *trans*-linalool oxide (furanoid) (3.275%), linalool (6.302%), (3*R*,6*S*)-2,2,6-trimethyl-6-vinyltetrahydro-2H-pyran-3-ol (2.787%) and phenethyl alcohol (6.760%). Notably, linalool, which is characterized by a citrus-like aroma, can be biosynthesized through the catalysis of geranyl diphosphate (GPP) by monoterpene synthases or released from glycosidic precursors during thermal processing (Fu et al., 2025). The ketone content was highest in the HAD and MD samples, accounting for 44.232% and 31.739% of the total volatiles, respectively. Representative ketones included 2,3-dihydro-3,5-dihydroxy-6-methyl-4H-pyran-4-one (HAD: 27.062%, MD: 19.543%), 2,5-dimethylfuran-3,4(2H,5H)-dione (HAD: 6.958%, MD: 0.598%), 1-(1H-pyrrol-2-yl)-ethanone (HAD: 3.572%, MD: 3.895%), 1-(2-furanyl)-ethanone (HAD: 2.352%, MD: 2.897%) and 4-cyclopentene-1,3-dione (HAD: 1.253%, MD: 1.752%). The results further indicate that heat treatment markedly reduced the content of ester compounds. This phenomenon, which has been widely reported in food systems, can be attributed to the susceptibility of esters to hydrolysis or thermal decomposition under heating conditions, resulting in the formation of smaller molecules such as carboxylic acids and alcohols, consequently, a decline in ester concentration (An et al., 2019).

**Fig. 6:**
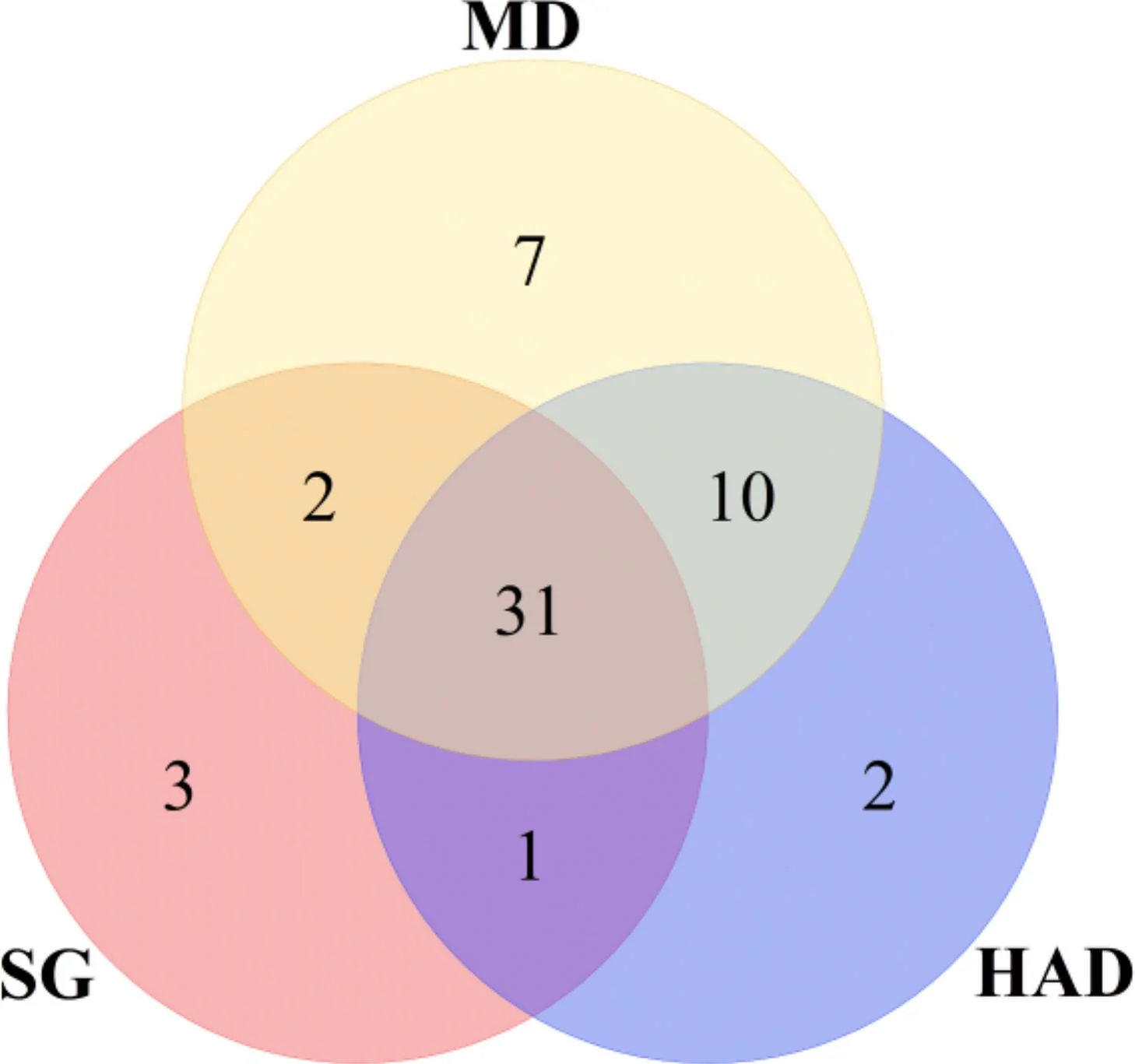
Venn diagram illustrating the distribution of tentatively identified VOCs detected by HS-SPME–GC–MS in SG, HAD, and MD. The numbers indicate the VOCs uniquely detected in or shared among the three sample groups.

**Fig. 7:**
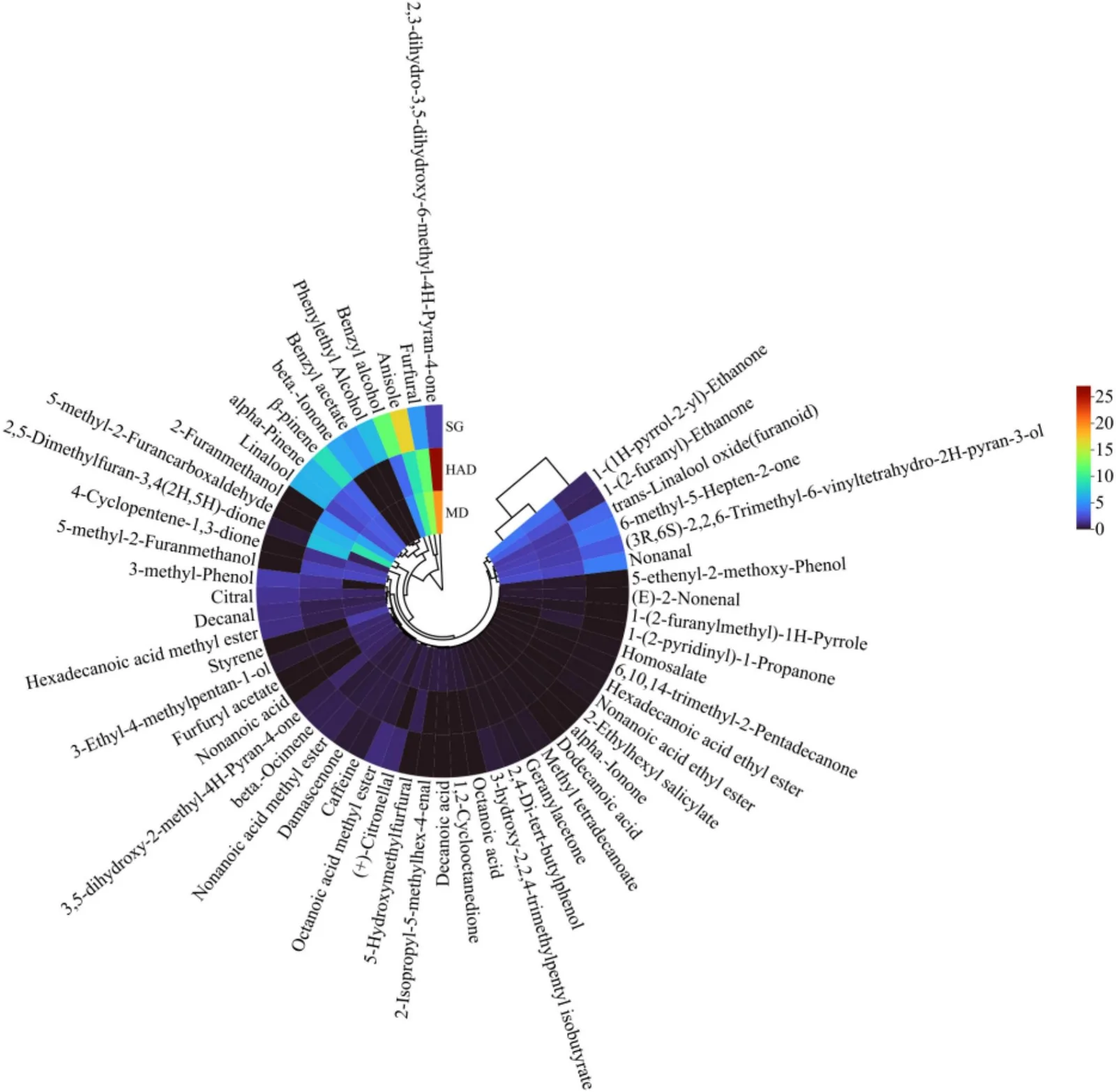
Hierarchical clustering heatmap showing the relative peak areas of tentatively identified VOCs determined by HS-SPME–GC–MS in SG, HAD, and MD. Rows represent individual VOCs, whereas columns represent samples. The color scale indicates the relative abundance of each VOC based on normalized peak areas, with warmer and cooler colors corresponding to higher and lower abundances, respectively.

The differences in the distribution of volatile compounds among the treatment groups are illustrated in Fig. 6. In total, 31 VOCs were identified across the three coffee peel samples, revealing clear group-specific compositional differences. The SG group contained three unique compounds—β-ionone, phenylethyl alcohol, benzyl acetate, whereas the MD group exhibited seven unique compounds, namely 2-isopropyl-5-methylhex-4-enal, 1,2-cyclooctanedione, furfuryl acetate, octanoic acid, nonanoic acid, decanoic acid, styrene. In contrast, only two components were uniquely detected in the HAD group: 1-(2-pyridinyl)-1-propanone and dodecanoic acid. The distribution of shared volatile components among sample groups was as follows: SG and MD samples contained two common compounds, namely (+)-citronellal and nonanoic acid ethyl ester; the SG and HAD samples shared 3-methylphenol, whereas the MD and HAD samples exhibited a greater number of shared compounds, including 10 compounds such as 5-methyl-2-furancarboxaldehyde, (E)-2-nonenal. These results demonstrate that different thermal processing treatment and processing methods markedly influence the composition of volatile compounds, thereby contributing to the diversification and enrichment of the overall flavor profile of coffee peel. Fig. 7 shows a cluster analysis heatmap based on the relative contents of the identified volatile compounds. As shown in Fig. 7, several compounds exhibited higher abundances and potential contributions to flavor in specific processing groups. Notably, 2,3-dihydro-3,5-dihydroxy-6-methyl-4H-pyran-4-one, furfural, 2-furanmethanol, 5-methyl-2-furanmethanol and 5-methyl-2-furancarboxaldehyde played pivotal roles in the MD and HAD groups, with their combined contents reaching 51.877% and 49.729%, respectively. In addition, 2,5-dimethylfuran-3,4(2H,5H)-dione was detected at a relatively high level (6.958%) in the HAD group, suggesting its potential importance as a flavor marker associated with hot-air thermal processing treatment. In the SG group, nonanal, anisole, 6-methyl-5-hepten-2-one and β-pinene were present at elevated levels, indicating their likely contributions to overall flavor profile. Among these, 6-methyl-5-hepten-2-one is mainly responsible for fresh green and leafy aromas (Xu et al., 2025), while β-pinene imparts characteristic fresh pine and herbal notes (Zhang et al., 2023).

### 3.3 Comprehensive analysis by GC–IMS and HS-SPME–GC–MS

**Fig. 8:**
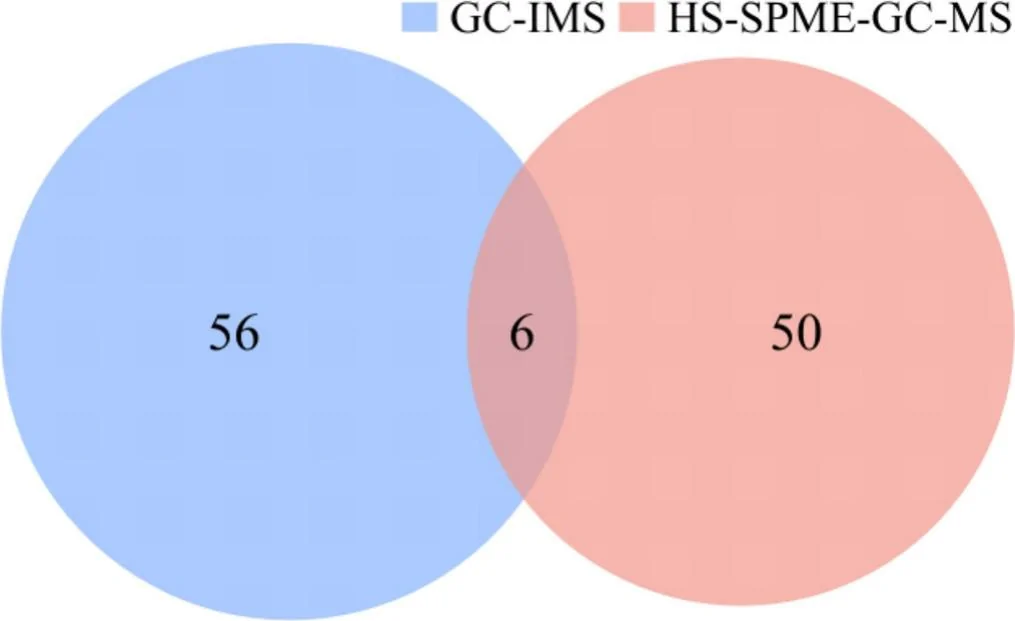
Venn diagram illustrating the comparison of tentative VOC assignments obtained by GC–IMS and HS-SPME–GC–MS after merging GC–IMS monomer and dimer signals. The numbers represent VOCs uniquely identified by each platform and those commonly detected by both analytical methods.

The VOCs detected by GC–IMS and HS-SPME–GC–MS were visualized using a Venn diagram after merging monomeric and dimeric signals (Fig. 8). Altogether, 112 VOCs were tentatively identified by the two analytical methods; however, only six compounds—furfural, 2-furanmethanol, alpha-pinene, 1-(2-furanyl)-ethanone, nonanal, and styrene—were detected by both techniques, indicating a marked divergence between the VOC profiles obtained. This limited overlap can be attributed to differences in the detection ranges and column polarities of the two methods (MXT-WAX for GC–IMS versus RTX-5MS for GC–MS). Specifically, GC–IMS exhibits higher sensitivity toward small, highly volatile compounds (C4–C10), whereas GC–MS is more suitable for detecting larger, semi-volatile compounds (C8–C20). Aroma composition is strongly influenced by origin and processing history, as shown in comparative volatile studies of hop varieties and their terroir-dependent profiles (Herkenhoff et al., 2024b). Consistent with this distinction, GC–IMS identified substantially more heterocyclic VOCs, particularly pyrazines and furans, than GC–MS, highlighting its superior sensitivity for small heterocyclic molecules (Nolvachai et al., 2023). In contrast, GC–MS demonstrates greater capability for the enrichment and identification of high-boiling-point volatile compounds (Li et al., 2024). Therefore, the combined application of GC–IMS and HS-SPME–GC–MS exploits the complementary advantages of both techniques, compensates for the limitations inherent to each individual method, and significantly expands the analytical coverage of VOCs in complex systems.

### 3.4 Differential volatile compounds in coffee peel subjected to different thermal processing treatment processes

**Fig. 9:**
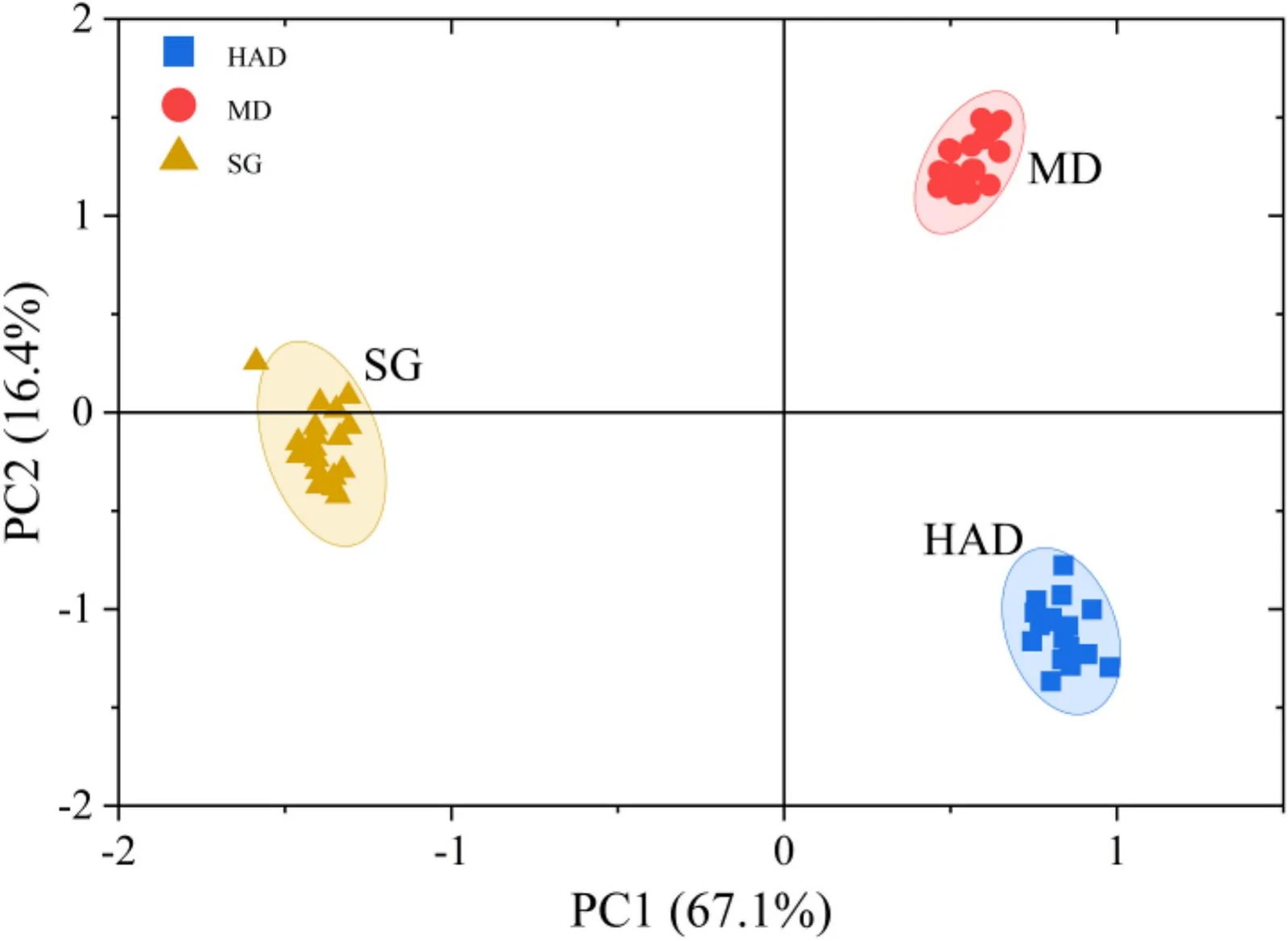
PCA score plot derived from normalized GC–IMS signal responses. PC1 and PC2 accounted for 67.1% and 16.4% of the total variance, respectively. SG, untreated sun-dried control; HAD, hot-air-dried treatment; MD, microwave-dried treatment.

**Fig. 10:**
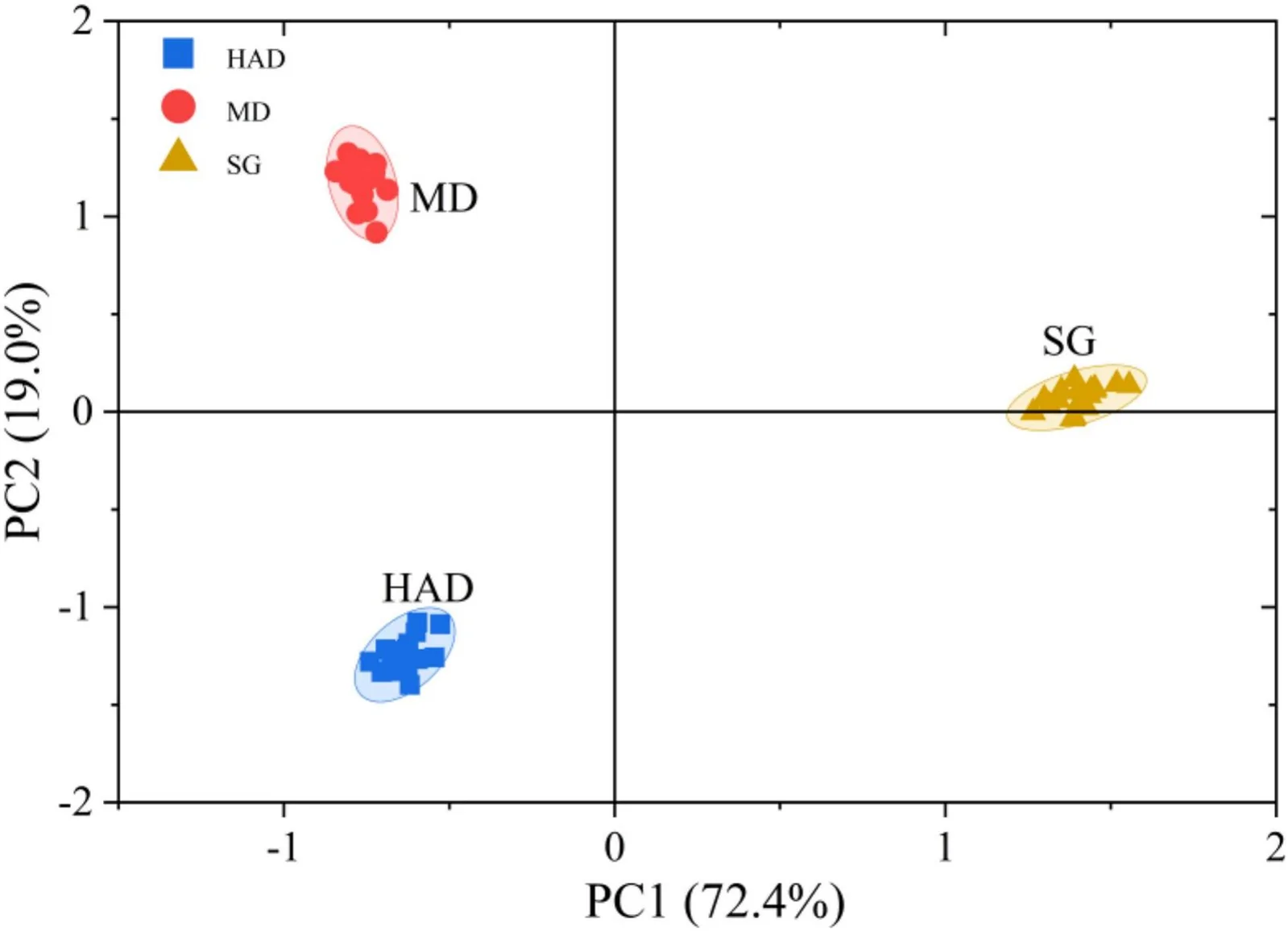
PCA score plot derived from HS-SPME–GC–MS relative peak-area data. PC1 and PC2 accounted for 72.4% and 19.0% of the total variance, respectively. SG, untreated sun-dried control; HAD, hot-air-dried treatment; MD, microwave-dried treatment.

Principal component analysis (PCA) is an unsupervised multivariate statistical method that reduces data dimensionality by transforming multiple correlated variables into a smaller number of principal components through linear combinations. To elucidate the differences in volatile compounds among coffee peel samples subjected to different processing treatments and the control group (sun-dried), PCA was performed separately on the volatile aroma datasets obtained by GC–IMS and HS-SPME–GC–MS. The corresponding results are presented in Fig. 9 and Fig. 10.

For the GC–IMS analysis, the first and second principal components accounted for 67.1% and 16.4% of the total variance, respectively, yielding a cumulative contribution rate of 83.5%. Similarly, in the HS-SPME–GC–MS analysis, the first two principal components explained 72.4% and 19.0% of the variance, respectively, with a cumulative contribution rate of 91.4%. These high cumulative variance contributions indicate that the first two principal components effectively represent the overall volatile flavor characteristics of the three coffee peel tea samples.

The PCA score plots generated from both GC–IMS and HS-SPME–GC–MS data showed clear spatial separation among the three sample groups, with relatively large intergroup distances, indicating significant differences in the composition and concentrations of volatile compounds among the treatments (Qi et al., 2025). Notably, the discrimination patterns obtained from the two analytical techniques were highly consistent, providing robust statistical evidence for evaluating the flavor quality differences of coffee peel subjected to different processing methods.

**Fig. 11:**
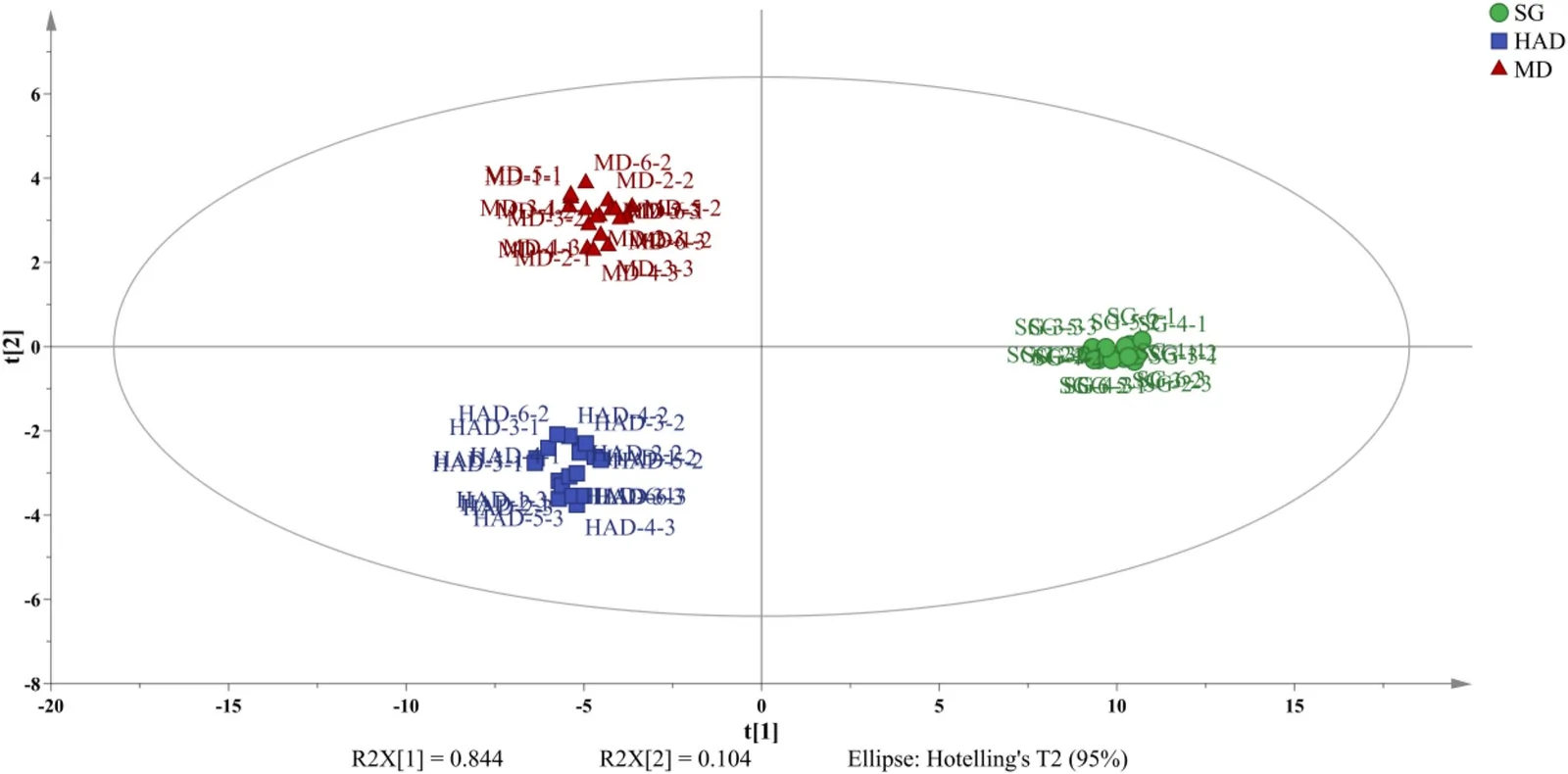
PLS–DA score plot derived from normalized GC–IMS signal responses. Each symbol represents an individual biological sample from SG, HAD, and MD, and the ellipse indicates the 95% confidence region of the PLS–DA model.

**Fig. 12:**
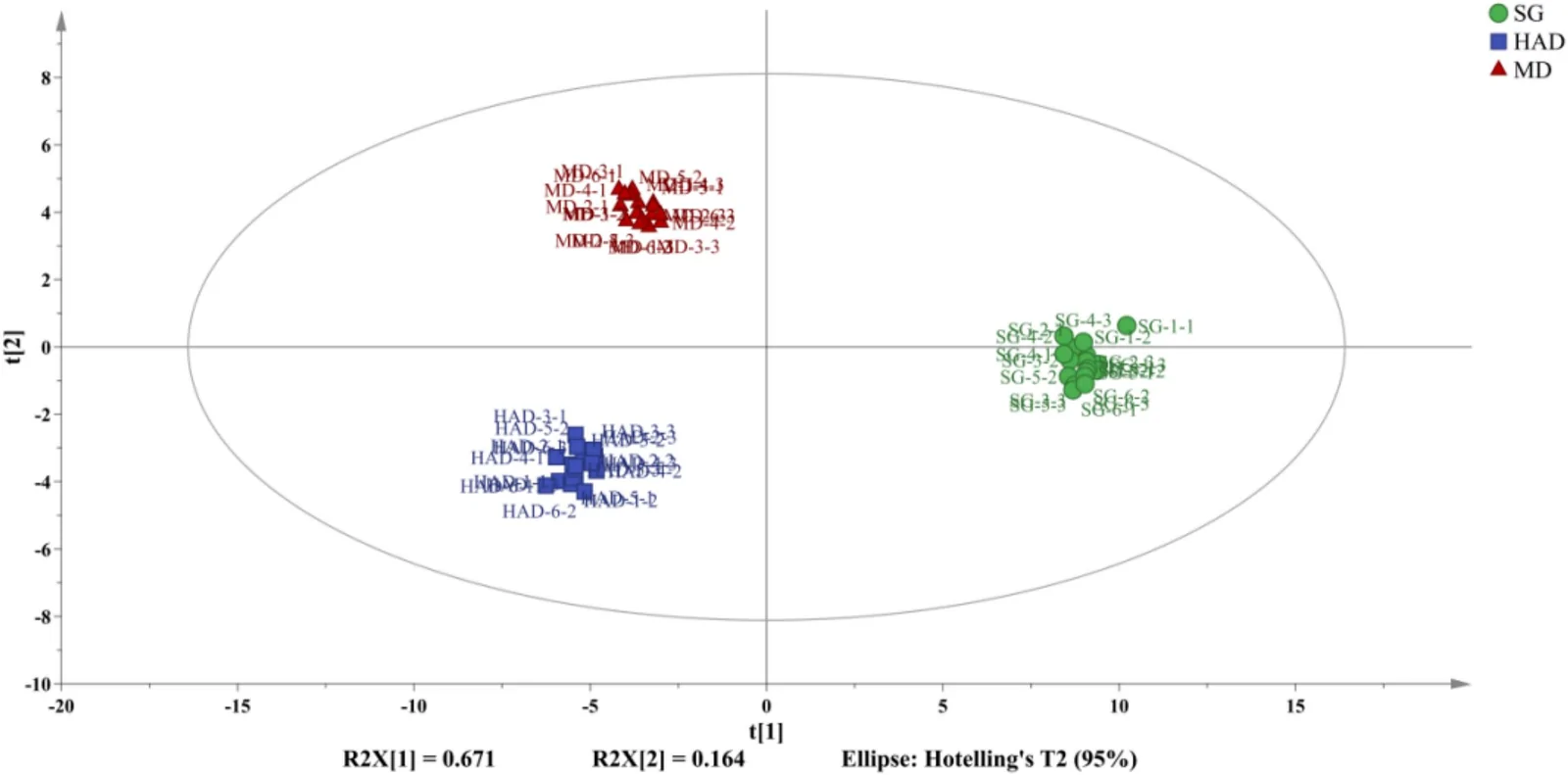
PLS–DA score plot derived from HS-SPME–GC–MS relative peak-area data. Each symbol represents an individual biological sample from SG, HAD, and MD, and the ellipse indicates the confidence region of the PLS–DA model.

PLS-DA is a supervised multivariate statistical analysis method that maximizes inter-group differences based on predefined classes (Y variables), thereby achieving better discrimination than PCA. In this study, the relative contents of volatile organic compounds (VOCs) detected by the two analytical techniques were used as dependent variables, while two processing treatments and the control group (sun-dried coffee peel) were used as independent variables to construct PLS-DA models (Fig. 11 and Fig. 12). Samples with different colors represent different treatment groups.

The PLS-DA discriminant models were constructed using MATLAB 2022b (MathWorks, USA). All samples were divided into a training set and a validation set at a ratio of 2:1 using the Kennard–Stone (KS) algorithm, and five-fold cross-validation was performed based on the training set. To evaluate the fitting performance and predictive robustness of the models, the coefficient of determination for calibration (R^2^_C_) was calculated using the training set, while the coefficient of determination for validation (Q^2^_v_) was obtained through leave-one-out cross-validation.

The model evaluation results showed that the prediction accuracies for both the training and validation sets reached 100% for the two datasets. For the GC–IMS data, the values of R^2^C and Q^2^v were 0.8805 and 0.8509, respectively. For the HS-SPME–GC–MS data, the corresponding values were 0.9481 and 0.9381, respectively. These results indicate that both models exhibited high and comparable R^2^C and Q^2^v values, demonstrating not only excellent fitting performance but also strong predictive capability without any risk of overfitting.

For the GC–IMS data, the model evaluation results showed that the explanatory abilities for the independent variables (R^2^X) and dependent variables (R^2^Y) were 0.987 and 0.989, respectively, while the predictive ability (Q^2^_v_) reached 0.975, likely reflecting the high sensitivity of GC–IMS toward trace VOCs (Yang et al., 2026). For the HS-SPME-GC–MS data, the PLS-DA model exhibited R^2^X and R^2^Y values of 0.997 and 0.998, respectively, with a Q^2^_v_ value of 0.994. To assess model robustness and predictive performance, a Q^2^ value greater than 0.5 is generally considered indicative of acceptable predictive ability, whereas a value above 0.9 reflects excellent predictive performance. Furthermore, the difference between R^2^Y and Q^2^ (R^2^Y − Q^2^) is widely used to evaluate potential overfitting, with values below 0.3 indicating a satisfactory balance between goodness of fit and predictive capability. In the present study, both models exceeded the Q^2^ > 0.9 criterion. The R^2^Y − Q^2^ values were 0.014 for the GC–IMS model and 0.004 for the HS-SPME–GC–MS model, both substantially below the recommended threshold of 0.3. These results indicate that the models achieved excellent explanatory and predictive performance while presenting a low risk of overfitting. The R^2^_c_ and Q^2^_v_ values of all models were close to 1, indicating excellent goodness-of-fit and strong predictive performance.

**Fig. 13:**
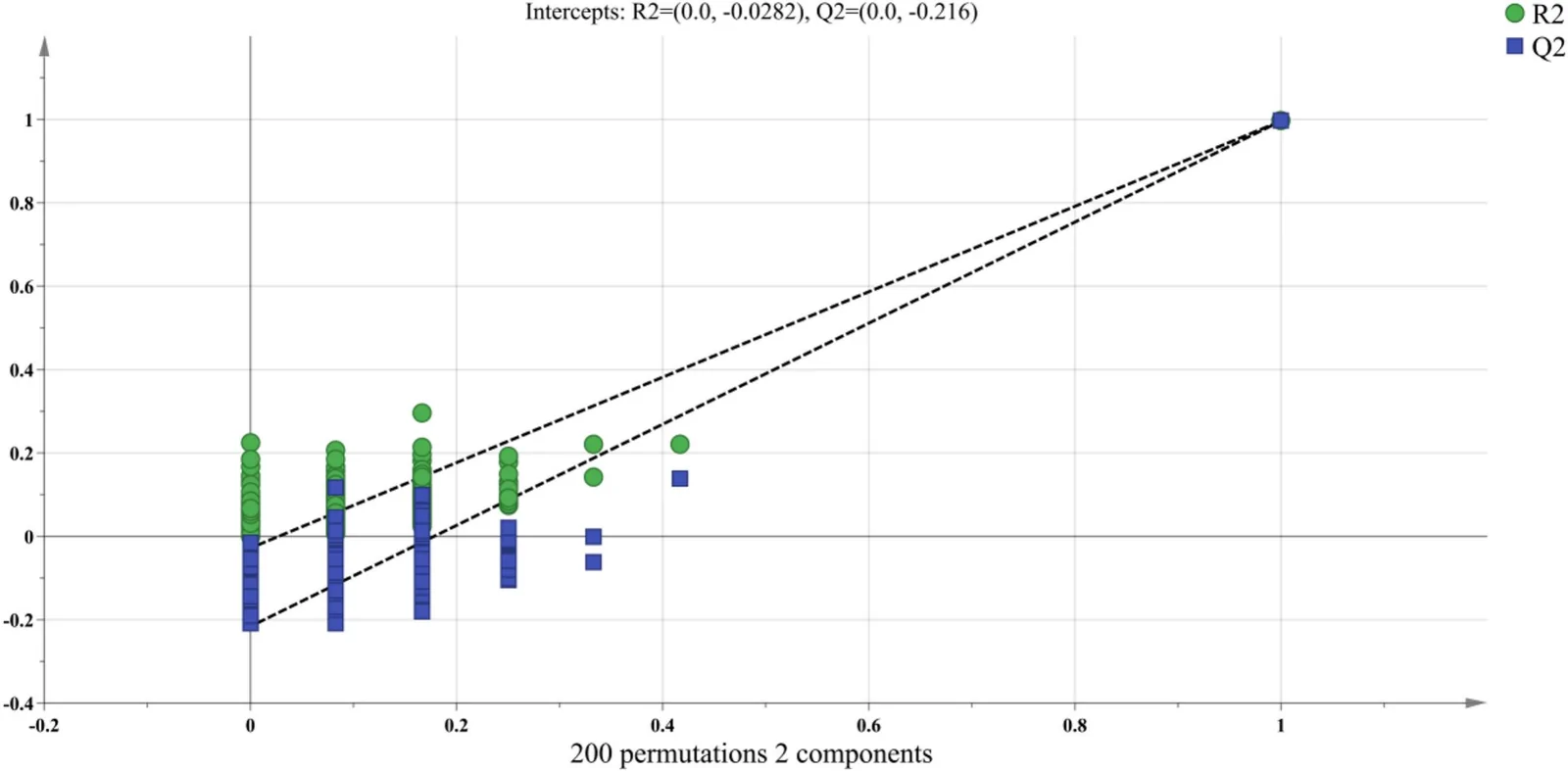
Permutation test (200 iterations) for validating the PLS–DA model constructed from normalized GC–IMS signal responses. The x-axis indicates the correlation between permuted and original class labels, while the corresponding R^2^ and Q^2^ values are displayed for each permutation.

**Fig. 14:**
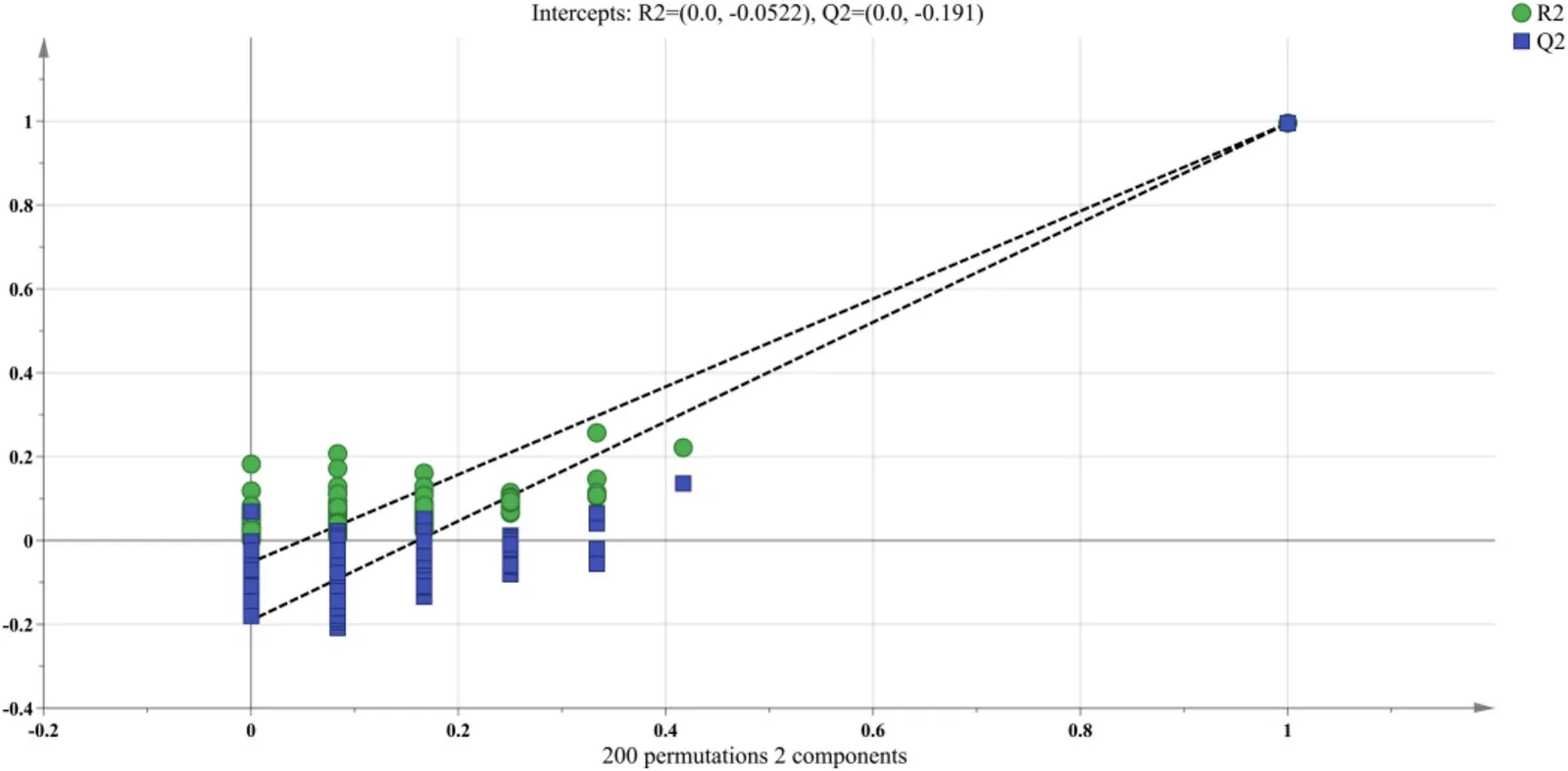
Permutation test (200 iterations) for validating the PLS–DA model constructed from HS-SPME–GC–MS relative peak-area data. The x-axis indicates the correlation between permuted and original class labels, while the corresponding R^2^ and Q^2^ values are displayed for each permutation.

Model reliability was further validated by 200-times permutation tests (Fig. 13 and Fig. 14). The intercepts of R^2^_c_ and Q^2^_v_ were −0.0282 and −0.216 for the GC–IMS model, and −0.0522 and −0.191 for the HS-SPME-GC–MS model, respectively. After permutation testing, both R^2^_c_ and Q^2^_v_ values decreased significantly, and all permuted Q^2^_v_ values were lower than the corresponding original values. Together with the statistical significance (*P* < 0.01), these results confirmed that neither model was overfitted, providing a reliable basis for the identification of differential VOCs (Liu et al., 2023; Qi et al., 2025).

**Fig. 15:**
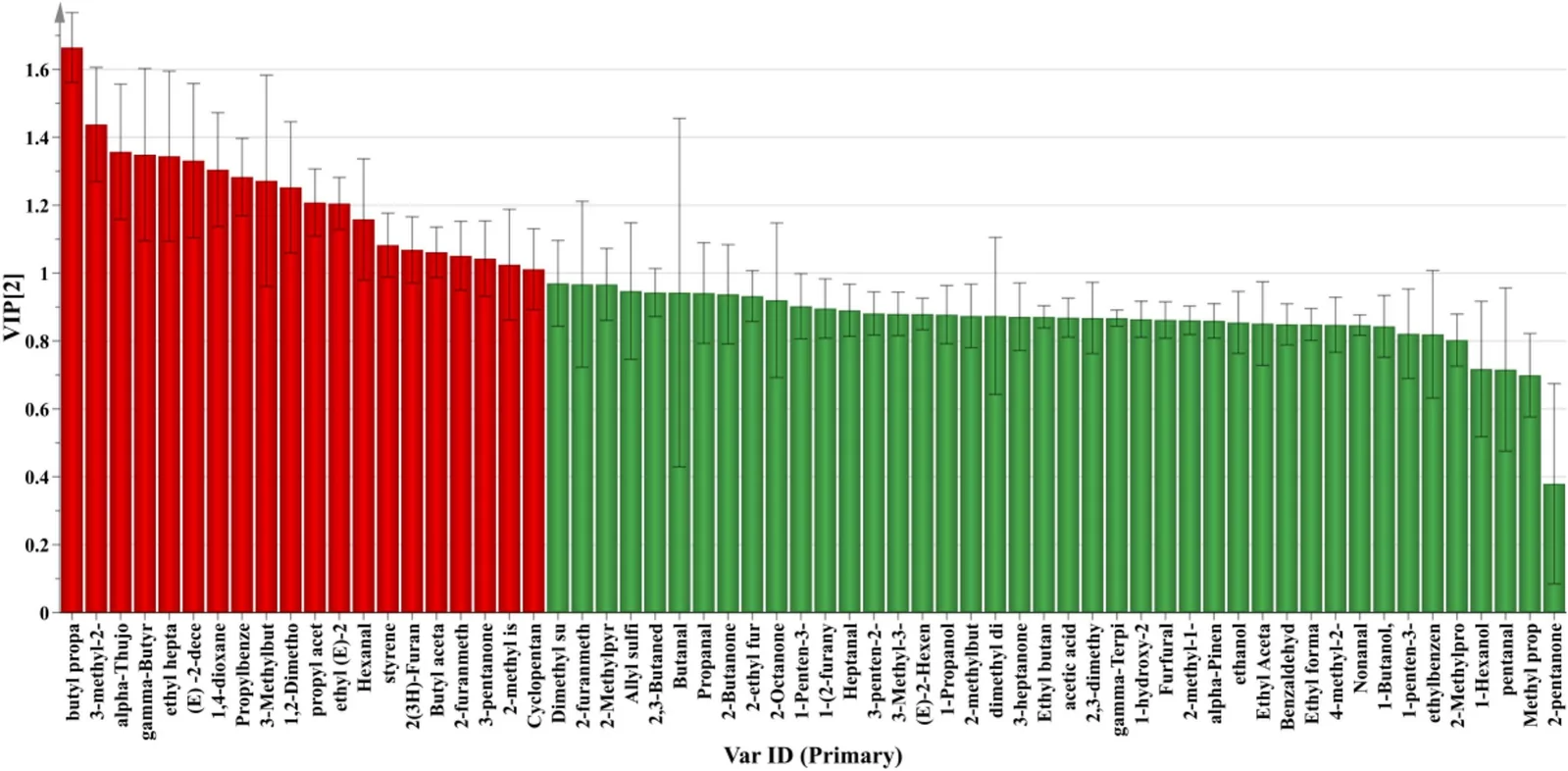
VIP scores obtained from the GC–IMS PLS–DA model. The y-axis indicates the VIP score, with variables exhibiting VIP > 1 considered key contributors to sample group separation. Error bars represent the uncertainty estimates provided by the statistical software.

**Fig. 16:**
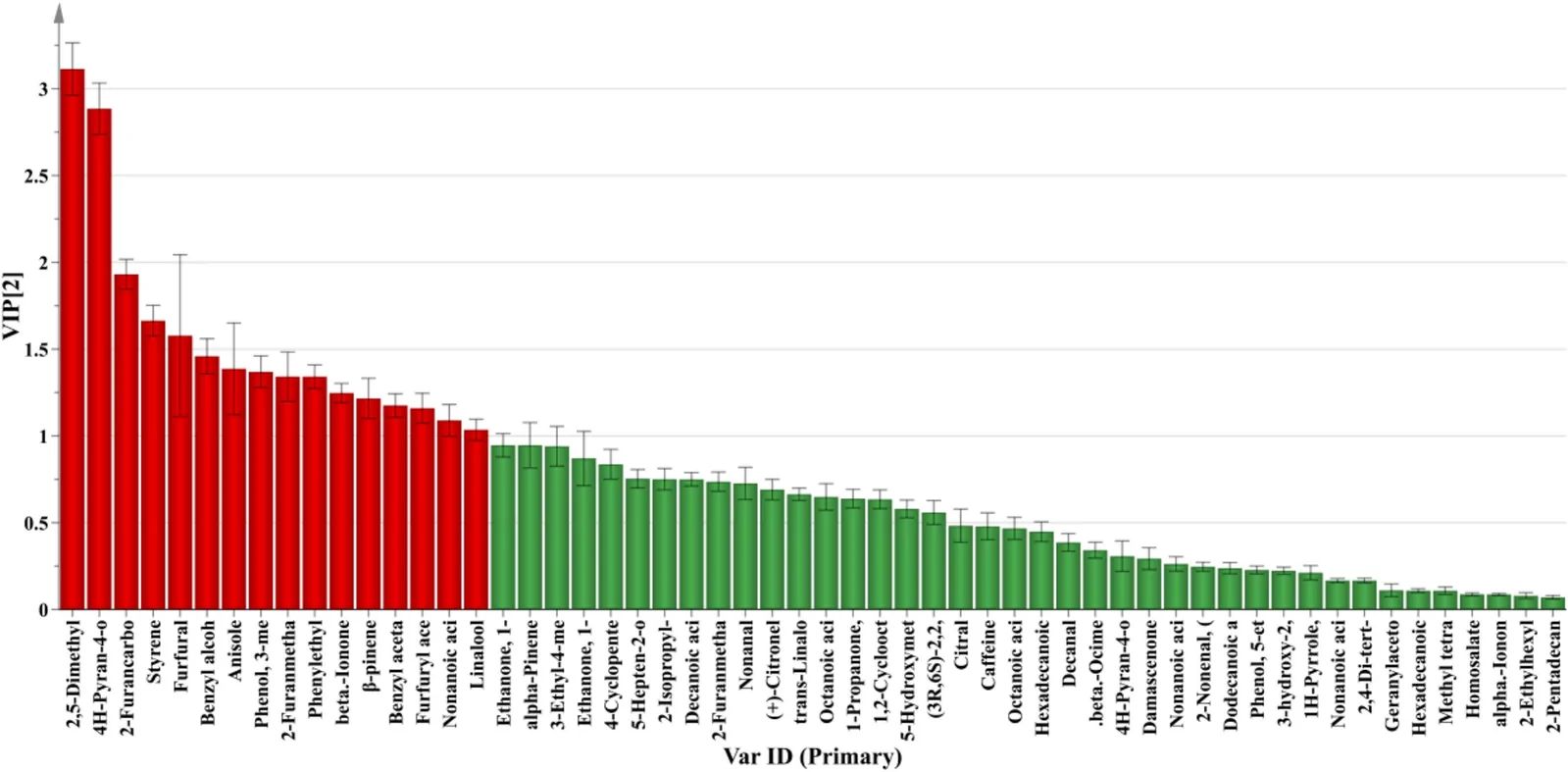
VIP scores obtained from the HS-SPME–GC–MS PLS–DA model. The y-axis indicates the VIP score, with variables exhibiting VIP > 1 considered key contributors to sample group separation. Error bars represent the uncertainty estimates provided by the statistical software.

According to the partial least squares discriminant analysis (PLS-DA), the variable importance in projection (VIP) score is commonly used to evaluate the relative contribution of individual variables to the statistical discrimination among sample groups. Using a threshold of VIP > 1, 20 compounds were preliminarily selected as potential discriminatory markers from the GC–IMS dataset, including butyl propanoate, 3-methyl-2-butanol, α-pinene ketone, γ-butyrolactone, ethyl heptanoate, (E)-2-decenal, 1,4-dioxane, propylbenzene, 3-methylbutanal, 1,2-dimethoxyethane, propyl acetate, ethyl (E)-2-butenoate, hexanal, styrene, 5-methyl-2(3H)-furanone, butyl acetate, 2-furanmethanethiol, 3-pentanone, 2-methylisoborneol, and cyclopentanone. Similarly, 16 compounds were preliminarily identified as potential discriminatory markers from the HS-SPME–GC–MS dataset, namely 2,5-dimethylfuran-3,4(2H,5H)-dione, 4H-pyran-4-one, 2,3-dihydro-3,5-dihydroxy-6-methyl-, 5-methyl-2-furfural, styrene, furfural, benzyl alcohol, anisole, *m*-cresol, furfuryl alcohol, 2-phenylethanol, β-ionone, β-pinene, benzyl acetate, furfuryl acetate, methyl nonanoate, and linalool (Figs. 15 and Fig. 16). These compounds were considered the potential discriminatory markers that contributed most to the classification of microwave-dried, hot-air-dried, and sun-dried coffee peel samples in the PLS-DA models.

### 3.5 Identification of key aroma components in coffee peel subjected to different thermal processing treatments

The actual contribution of volatile compounds to a sample's flavor profile depends not only on their relative abundance but also on a comprehensive evaluation in conjunction with the flavor thresholds of individual substance (Zhang et al., 2019). The aroma characteristics of foods are primarily determined by key compounds with high ROAVs. The contribution of individual volatile compound to the overall flavor profile is positively correlated with its ROAV. Generally, compounds with ROAV ≥1 are considered to make a key contribution to the sample's overall flavor and are defined as key flavor compounds. Those with 0.1 ≤ ROAV <1 are regarded as having a modifying effect on the overall flavor and are classified as modifying flavor compounds. In contrast, compounds with ROAV <0.1 are considered to have a negligible impact on the overall flavor (Yue et al., 2025).

The potential contribution of volatile organic compounds (VOCs) to the overall aroma profile was preliminarily evaluated using the relative odor activity value (ROAV) approach (Sun et al., 2026), which integrates odor threshold values with the relative abundances determined by HS-SPME-GC–MS and the relative peak areas obtained from GC–IMS. In addition, compounds consistently detected across samples and possessing exceptionally low odor thresholds were also considered as potential aroma-active candidates (Chen et al., 2026). Within the GC–IMS dataset, 2-furanmethanethiol, which was detected in all three samples (SG, HAD, and MD), exhibited an extremely low literature-reported odor threshold (0.00008 mg/kg) and was therefore regarded as a compound of particular interest. Likewise, based on the combined HS-SPME–GC–MS and GC–IMS results, β-damascenone (odor threshold: 0.000007 mg/kg), detected exclusively in the SG sample, and benzyl alcohol (odor threshold: 0.0001 mg/kg), detected in all three groups, were also identified as potential aroma-active compounds with high sensory relevance. These three compounds were selected as reference compounds, and the highest ROAV value (ROAVmax) was assigned a value of 100. The ROAV values of the remaining compounds were then calculated relative to this reference, and the results are summarized in Table 1.

**Table 1:** ROAV of aroma-active compounds of different coffee peels.

VOCs	Odor threshold(mg/kg)	Aroma properties	ROAV/%		
			SG	HAD	MD
1-Hexanol	0.0056	Fruity	3.23	1.74	1.44
3-Methyl-1-butanol	0.004	Fruity	4.16	0.51	0.48
4-Methyl-2-pentanol	2.5	Sour/Fermented	0.00	0.00	1.09
2-Furanmethanethiol	0.00008	Roasted	100.00	100.00	100.00
2-Octanone	0.0502	Other	0.79	0.27	0.17
2,3-Butanedione	0.00774	Other	3.04	0.29	0.37
3-Heptanone	0.08	Fruity	0.22	0.04	0.03
2-Pentanone	1.38	Fruity	0.01	0.00	0.97
1-Hydroxy-2-propanone	10	Sweet	0.01	0.83	0.00
1-Penten-3-one	0.023	Spices	1.03	0.18	0.16
Ethyl acetate	0.005	Fruity	2.82	0.24	0.26
Butyl acetate	0.058	Fruity	0.08	0.19	0.08
Ethyl butanoate	0.0009	Fruity	8.27	7.26	4.74
Ethyl heptanoate	0.0019	Fruity	1.00	0.61	0.22
2-Methylbutanoic acid ethyl ester	0.000063	Fruity	98.62	15.62	11.15
nonanal	0.0011	Floral	16.68	6.93	6.21
(E)-2-decenal	0.0003	Other	47.93	12.04	13.42
3-Methylbutanal	0.0011	Nutty/Cocoa	56.35	15.35	14.27
Benzaldehyde	0.32	Nutty/Cocoa	1.31	0.15	0.13
Heptanal	0.0028	Green/Vegetative	7.19	0.62	0.75
Hexanal	0.005	Green/Vegetative	4.33	0.58	0.74
2-Methylpropanal	0.0015	Green/Vegetative	21.25	9.05	6.53
Pentanal	0.012	Sour/Fermented	0.89	0.18	0.15
Propanal	0.0151	Sour/Fermented	0.59	0.08	0.09
Butanal	0.000182	Nutty/Cocoa	55.49	12.60	11.09
(E)-2-hexenal	0.0887	Green/Vegetative	0.55	0.03	0.04
Styrene	0.065	Other	0.06	0.16	0.06
Alpha-pinene	0.014	Other	2.88	3.99	3.26
Dimethyl disulfide	0.0011	Green/Vegetative	18.53	5.12	4.43
Allyl sulfide	0.1	Other	0.23	0.06	0.05
Dimethyl sulfide	0.00012	Green/Vegetative	36.71	18.19	11.14
(E)-2-nonenal	0.00019	Green/Vegetative	0.00	3.07	2.52
Decanal	0.003	Fruity	0.00	0.46	0.36
Damascenone	0.000126	Floral	0.21	8.29	4.87
Β-ionone	0.000007	Floral	100.00	–	–
Benzyl alcohol	0.0001	Floral	14.21	100.00	100.00
Linalool	0.00022	Floral	3.45	26.90	23.88
3-Methylphenol	0.031	Other	0.01	0.11	–
Anisole	0.1775	Other	0.01	0.15	0.12

Note: Odor thresholds were obtained from literature (Gemert, 2003). All odor thresholds obtained from this reference were measured in water, and for each substance, the measurement value from the most recent year is adopted. Aroma descriptions are from http://www.perflavory.com/search.php. In the table, “–” indicates that the substance was not detected, and “0.00” indicates that the ROAV value of the substance is too low.

In total, more than 39 aroma-active compounds (with ROAV ≥0.1) were tentatively identified across the samples. Among the three coffee-husk samples (SG, HAD, and MD), 23, 26, and 24 compounds, respectively, exhibited ROAV values ≥1. Although the HAD sample contained the largest number of volatiles with ROAV ≥1, their values were relatively evenly distributed. In contrast, the SG sample contained fewer potential aroma compounds, but the high-ROAV compounds were more concentrated, resulting in the greatest overall aroma intensity.

In the SG sample, the major potential aroma-active compounds ranked according to their ROAV values were estimated to be β-ionone (100), 2-furanmethanethiol (100), 2-methylbutanoic acid ethyl ester (98.62), 3-methylbutanal (56.35), butanal (55.49), (E)-2-decenal (47.93), dimethyl sulfide (36.71), 2-methylpropanal (21.25), dimethyl disulfide (18.53), benzyl alcohol (14.21), and nonanal (16.68). Among these compounds, β-ionone and 2-furanmethanethiol exhibited the highest ROAV values (both 100), suggesting that they may represent the predominant contributors to the intense floral, fruity, and roasted aroma characteristics of the SG samples.

The putative aroma-active compounds identified in the HAD sample were 2-furanmethanethiol (100), benzyl alcohol (100), linalool (26.90), dimethyl sulfide (18.19), 2-methylbutanoic acid ethyl ester (15.62), 3-methylbutanal (15.35), butanal (12.60), (E)-2-decenal (12.04), 2-methylpropanal (9.05), damascenone (8.29), nonanal (6.93), and dimethyl disulfide (5.12). Notably, the ROAV value of damascenone in the HAD sample reached 8.29, which was substantially higher than those observed in the SG (0.21) and MD (4.87) samples. This result suggests that hot-air drying may promote the formation or retention of compounds associated with fruity and honey-like aroma characteristics.

In the MD sample, the principal putative aroma-active compounds included 2-furanmethanethiol (100), benzyl alcohol (100), linalool (23.88), 3-methylbutanal (14.27), (E)-2-decenal (13.42), 2-methylbutanoic acid ethyl ester (11.15), dimethyl sulfide (11.14), butanal (11.09), 2-methylpropanal (6.53), damascenone (4.87), and dimethyl disulfide (4.43). In addition, (E)-2-nonenal was detected exclusively in the HAD and MD samples, with ROAV values of 3.07 and 2.52, respectively, indicating that it may be a potential contributor to the grassy and fatty aroma notes of these samples.

The ROAV values of 2-furanmethanethiol and benzyl alcohol were both markedly higher in the HAD and MD samples than in the SG samples, particularly for benzyl alcohol, whose ROAV value was only 14.21 in the SG samples. These findings suggest that hot-air drying and microwave drying may promote the formation and retention of benzyl alcohol and sulfur-containing furan compounds, thereby enhancing potential coffee-like, roasted, caramel-like, and smoky aroma notes in the processed samples. In contrast, the SG samples retained relatively higher levels of compounds potentially associated with fruity and green aroma characteristics, including ethyl 2-methylbutyrate, 3-methylbutanal, butanal, and dimethyl sulfide, resulting in a more balanced and complex overall aroma profile.

To more accurately identify potential key aroma-contributing compounds, volatile compounds that simultaneously met the criteria of a variable importance in projection (VIP) score ≥ 1 and a relative odor activity value (ROAV) ≥ 1 were screened and comparatively analyzed. Based on this integrated evaluation, eight volatile compounds—2-furanmethanethiol, 3-methylbutanal, (E)-2-decenal, ethyl heptanoate, hexanal, 2-phenylethanol, linalool, and β-ionone—were preliminarily identified as potential aroma markers of coffee peel subjected to different drying treatments. Nevertheless, further confirmation of their aroma contributions requires gas chromatography–olfactometry (GC–O) combined with sensory evaluation.

**Fig. 17:**
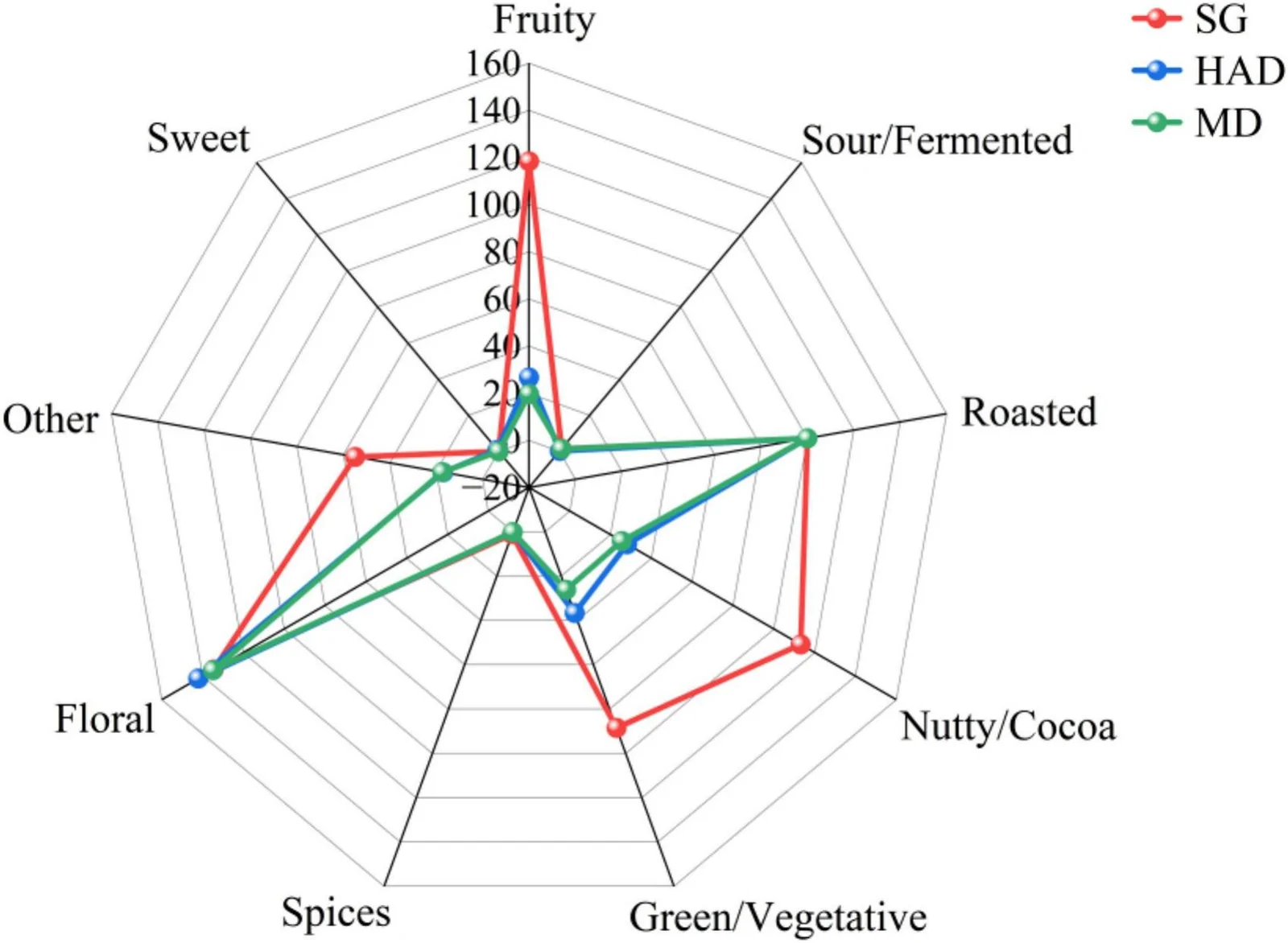
Radar chart illustrating the estimated aroma profiles of coffee peel samples (SG, HAD, and MD) based on ROAV values across nine aroma categories, including herbaceous/vegetative, acidic/fermented, fruity, floral, sweet, nutty/cocoa, spicy, roasty, and other aroma attributes.

According to the flavor classification system established by the Specialty Coffee Association (SCA), nine core aroma categories from the Coffee Taster's Flavor Wheel—herbaceous/vegetative, acidic/fermented, fruity, floral, sweet, nutty/cocoa, spicy, roasty, and others—were adopted as putative aroma attributes for coffee peels. Using the sun-dried samples (SG) as the reference, the overall aroma characteristics of coffee peels subjected to hot-air drying (HAD) and microwave drying (MD) were systematically compared. Based on the quantitative results of the key aroma-active compounds (Table 1), radar plots illustrating the estimated aroma profiles of coffee peel under different drying treatments were constructed (Fig. 17).

The results demonstrated that the three coffee cascara samples exhibited distinct putative aroma characteristics. Overall, the sweetness, acidity/fermentation, and spicy dimensions showed relatively low response intensities. Specifically, the ROAV values for the sweetness dimension were 0.01 (SG), 0.83 (HAD), and 0.00 (MD); for acidity/fermentation, the corresponding values were 1.48 (SG), 0.26 (HAD), and 1.33 (MD); and for the spicy dimension, the ROAV values were 1.03 (SG), 0.18 (HAD), and 0.16 (MD). In contrast, the roasty attribute exhibited identical intensity across all three samples (ROAV = 100), indicating that neither hot-air drying nor microwave drying substantially altered the roasted putative aroma characteristics of coffee cascara.

Notably, the SG samples displayed markedly higher intensities in the fruity (ROAV = 118.41), nutty/cocoa (ROAV = 113.15), and herbaceous/vegetable (ROAV = 88.56) dimensions. Consequently, the SG sample exhibited the largest polygonal area and the fullest contour in the radar chart, suggesting that traditional sun drying promotes the retention and synergistic enrichment of diverse volatile compounds, thereby generating a rich, balanced, and multi-layered putative aroma profile.

The HAD samples were characterized by the most prominent putative floral attribute (ROAV = 142.12), which formed a distinctive sharp peak in the radar chart. This result indicates that hot-air drying may favor the formation or enrichment of floral aroma compounds. In contrast, the MD samples exhibited relatively lower estimated intensities across most aroma dimensions, except for the putative roasty attribute, which was comparable to that of the SG samples. The estimated ROAV values for the putative fruity, nutty/cocoa, and herbaceous/vegetative aroma attributes of the MD samples were 19.73, 25.49, and 26.15, respectively. Among the evaluated aroma categories, only the putative floral attribute of the MD samples (ROAV = 134.96) was comparable to that of the SG samples.

In summary, the SG samples exhibited a more complex estimated aroma profile, dominated by putative herbaceous/vegetative, fruity, and nutty/cocoa aroma attributes. In contrast, the HAD samples were distinguished by a pronounced putative floral aroma attribute, whereas the MD samples were primarily characterized by putative floral and roasty aroma attributes. This gradient pattern was generally consistent with the clustering results obtained from the GC–IMS and HS-SPME–GC–MS analyses, suggesting that different drying methods may modulate the overall aroma profile of coffee peel. In particular, the drying treatments appeared to influence the relative enrichment or depletion of compounds associated with the putative fruity, floral, nutty/cocoa, and herbaceous/vegetative aroma attributes, whereas the estimated roasty attribute remained relatively stable across the different drying methods.

### 3.6 Discussion of formation mechanisms

During the thermal processing process of coffee cascara, the formation of volatile flavor compounds is mainly governed by three major pathways: the Maillard reaction (including Strecker degradation), lipid oxidation and thermal degradation, and caramelization. These reactions occur simultaneously and interact with one another, collectively shaping the distinctive aroma characteristics of cascara tea.

The Maillard reaction and Strecker degradation represent the most critical pathways involved in aroma formation during coffee cascara thermal processing. Coffee cascara is naturally rich in reducing sugars, with total sugar contents reaching up to 19.3%, as well as a wide variety of amino acids, among which 17 types have been identified. These constituents provide abundant precursors for Maillard reactions. During thermal processing, the carbonyl groups of reducing sugars react with the amino groups of amino acids through condensation reactions, followed by Amadori rearrangement, generating a series of volatile intermediates and products, including α-dicarbonyl compounds, furans, and pyrazines.

Simultaneously, the generated α-dicarbonyl compounds further participate in Strecker degradation reactions with amino acids, leading to oxidative decarboxylation and the formation of aldehydes containing one fewer carbon atom than their precursor amino acids. For instance, leucine is converted into 3-methylbutanal, which contributes malty and cocoa-like aromas; methionine produces sulfur-containing compounds associated with sulfurous and meaty notes; and proline reacts with sugars to generate 2-acetyl-1-pyrroline, imparting nutty and roasted aromas. Variations in the concentrations of these Strecker aldehydes and pyrazines are therefore considered key factors underlying the pronounced differences in roasted, sweet, and nutty aroma characteristics among coffee cascara teas produced using different primary processing methods, such as natural, washed, and honey processing.

Lipid oxidation and thermal degradation also play important roles in the formation of aroma compounds during coffee cascara thermal processing. To date, fifteen fatty acids have been identified in coffee cascara, with a total lipid content reaching up to 6.75%, among which unsaturated fatty acids constitute a substantial proportion. Under high-temperature and aerobic conditions, these unsaturated fatty acids readily undergo autoxidation, leading to the formation of hydroperoxides that subsequently decompose into low-molecular-weight volatile compounds, including aldehydes (such as hexanal, nonanal, and (E)-2-decenal), ketones, and short-chain hydrocarbons. These lipid-derived volatiles typically contribute grassy, fatty, and fruity aroma characteristics to cascara tea.

In addition, lipids can undergo direct thermal degradation, producing free fatty acids as well as small-molecule aldehydes and ketones. In particular, honey-processed coffee cascara tea exhibited relatively high levels of (E)-2-decenal and other unsaturated aldehydes, suggesting that lipid oxidation contributes more prominently to aroma formation under this processing condition. Furthermore, the carbonyl compounds generated through lipid oxidation may also participate in the Maillard reaction network, thereby indirectly promoting the formation of nitrogen-containing heterocyclic compounds, such as pyrazines.

Caramelization and other thermal degradation reactions also constitute important pathways in the formation of aroma compounds during coffee cascara thermal processing. The abundant reducing sugars present in coffee cascara undergo dehydration, fragmentation, and condensation reactions under high-temperature conditions, leading to the formation of furans such as furfural and 5-hydroxymethylfurfural, as well as caramel-like pigments. These furan compounds not only directly impart caramel-like and sweet aroma characteristics but also act as important intermediates in subsequent Maillard reactions. Previous studies have demonstrated that furans generated through caramelization are often among the most abundant volatile compounds formed during coffee thermal processing treatment.

In addition, processing conditions and substrate composition are known to influence volatile evolution and flavor complexity in related fermentation systems (Praia et al., 2022). For instance, phenolic acids naturally abundant in coffee cascara, such as chlorogenic acid, can undergo thermal degradation to produce volatile phenolic compounds including guaiacol and 4-vinylguaiacol. These compounds contribute smoky, spicy, and phenolic aroma notes, thereby further enhancing the complexity of cascara tea aroma profiles.

## 4 Conclusion

In this study, a dual-platform analytical approach was employed to comparatively evaluate the effects of microwave drying (MD), hot-air drying (HAD), and traditional sun drying (SG) on the volatile composition of coffee peel. Gas chromatography–ion mobility spectrometry (GC–IMS) and headspace solid-phase microextraction coupled with gas chromatography–mass spectrometry (HS-SPME–GC–MS) demonstrated strong complementarity in the analysis of volatile organic compounds (VOCs). Specifically, GC–IMS exhibited greater sensitivity toward low-molecular-weight aldehydes, ketones, and certain heterocyclic compounds, whereas HS-SPME–GC–MS showed superior performance in the extraction and tentative identification of medium-molecular-weight alcohols, esters, and long-chain aldehydes. By integrating the results from both analytical platforms, a total of 112 VOCs were tentatively identified, although only six compounds were detected by both methods. This limited overlap indicates that a single analytical technique may be insufficient to comprehensively characterize the volatile aroma profile of coffee peel, whereas the integration of complementary analytical platforms can substantially expand the analytical coverage and dimensionality of volatile aroma characterization.

Based on the combined screening criteria of a variable importance in projection (VIP) score > 1 and a relative odor activity value (ROAV) ≥ 1, eight compounds—2-furanmethanethiol, 3-methylbutanal, (E)-2-decenal, ethyl heptanoate, hexanal, 2-phenylethanol, linalool, and β-ionone—were preliminarily proposed as potential differential aroma markers associated with processing-related flavor differences. Further estimation based on ROAV-derived aroma attributes suggested distinct putative aroma profiles among the three drying treatments. The SG samples appeared to exhibit a relatively complex estimated aroma profile, characterized primarily by putative herbaceous/vegetative, fruity, and nutty/cocoa aroma attributes. This pattern may be associated with the mild thermal conditions during sun drying, which could facilitate the preservation of thermolabile compounds such as terpenoids and lactones while allowing continued enzymatic activity. In contrast, the HAD samples exhibited a particularly pronounced putative floral aroma attribute, possibly because hot-air drying promoted the hydrolysis of glycosidically bound aroma precursors and enhanced Strecker degradation, thereby increasing the abundance of floral-related compounds such as linalool, β-damascenone, and 2-phenylethanol. Meanwhile, the MD samples appeared to combine putative floral and roasty aroma attributes. The rapid internal heating induced by microwave drying may accelerate the Maillard reaction and caramelization, leading to the formation of furans, pyrazines, and sulfur-containing heterocyclic compounds that may contribute to putative caramel-like, nutty, and smoky aroma notes.

Notably, based on the ROAV estimation, the roasty attribute exhibited an identical value of 100 across all three sample groups, tentatively suggesting that this aroma dimension may be relatively insensitive to thermal processing treatment method within the limits of this estimation approach. This phenomenon might be related to the intrinsic thermal degradation behaviour of native precursors in coffee peel, such as chlorogenic acid and trigonelline.

Overall, the findings of this study provide valuable insights for the preliminary selection of drying technologies and the flavor-oriented development of coffee peel tea products. However, the actual contributions of the identified putative aroma markers to the perceived aroma profile, together with their dynamic transformation mechanisms during processing, remain to be fully elucidated. Future research should integrate trained sensory panel evaluations with advanced analytical approaches, including gas chromatography–olfactometry–mass spectrometry (GC–O–MS) and stable isotope labeling, to validate the sensory relevance of these candidate markers and further clarify the formation pathways and interaction mechanisms of aroma-active compounds in coffee peel products.

## CRediT authorship contribution statement

**Congyan Meng:** Writing – review & editing, Writing – original draft, Visualization, Methodology, Investigation, Conceptualization. **Jie Wu:** Methodology, Investigation. **Chunrong Yan:** Visualization. **Jianqiang Wei:** Supervision. **Yuanxing Zhang:** Software, Investigation. **Tianjun Yuan:** Writing – review & editing, Formal analysis. **Ying Hou:** Writing – review & editing, Supervision, Funding acquisition, Conceptualization.

## Declaration of competing interest

The authors declare that they have no known competing financial interests or personal relationships that could have appeared to influence the work reported in this paper.

## Data Availability

Data will be made available on request.

## Appendix A Supplementary data

Supplementary material 1 Coffee peel volatiles: classification by VIP≥1 and ROAV≥1.

Supplementary material 2 Table of traceable odour-threshold source.

Supplementary material 3 GC-IMS analysis results.

Supplementary material 4 HS-SPME-GC-MS analysis results.
