# Supplementary material for: Analysis of characteristic flavor compounds in coffee peels subjected to different thermal processing treatments based on GC–IMS and HS-SPME–GC–MS combined with chemical pattern recognition
Source: Food Chem X. 2026 Aug 10;38:104256. doi: 10.1016/j.fochx.2026.104256 (PMC13524620; doi:10.1016/j.fochx.2026.104256)
Supplement: Supplementary file 1 [file mmc1.docx]

Table S1. Classification of volatile compounds identified in coffee peel samples based on VIP (VIP≥1) and ROAV (ROAV≥1) criteria

| **VOCs** | **VIP＞1** | **ROAV≥1** | **Category** |
| --- | --- | --- | --- |
| Butyl propanoate | yes | no | Differential markers only |
| 3‑methyl‑2‑butanol | yes | no | Differential markers only |
| α‑thujone | yes | no | Differential markers only |
| γ‑butyrolactone | yes | no | Differential markers only |
| ethyl heptanoate | yes | yes | Intersection (core putative markers) |
| (E)‑2‑decenal | yes | yes | Intersection (core putative markers) |
| 1,4‑dioxane | yes | no | Differential markers only |
| propylbenzene | yes | no | Differential markers only |
| 3‑methylbutanal | yes | yes | Intersection (core putative markers) |
| 1,2‑dimethoxyethane | yes | no | Differential markers only |
| propyl acetate | yes | no | Differential markers only |
| ethyl (E)‑2‑butenoate | yes | no | Differential markers only |
| hexanal | yes | yes | Intersection (core putative markers) |
| styrene | yes | no | Differential markers only |
| 5‑methyl‑2(3H)‑furanone | yes | no | Differential markers only |
| butyl acetate | yes | no | Differential markers only |
| 2‑furanmethanethiol | yes | yes | Intersection (core putative markers) |
| 3‑pentanone | yes | no | Differential markers only |
| 2‑methylisoborneol | yes | no | Differential markers only |
| cyclopentanone | yes | no | Differential markers only |
| 2,5‑dimethylfuran‑3,4(2H,5H)‑dione | yes | no | Differential markers only |
| 4H‑pyran‑4‑one,2,3‑dihydro‑3,5‑dihydroxy‑6‑methyl‑ | yes | no | Differential markers only |
| 5‑methyl‑2‑furancarboxaldehyde | yes | no | Differential markers only |
| styrene | yes | no | Differential markers only |
| furfural | yes | no | Differential markers only |
| benzyl alcohol | yes | yes | Intersection (core putative markers) |
| anisole | yes | no | Differential markers only |
| 3‑methylphenol | yes | no | Differential markers only |
| 2‑furanmethanol | yes | no | Differential markers only |
| phenylethyl alcohol | yes | no | Differential markers only |
| β-Ionone | yes | yes | Intersection (core putative markers) |
| β‑pinene | yes | no | Differential markers only |
| benzyl acetate | yes | no | Differential markers only |
| furfuryl acetate | yes | no | Differential markers only |
| nonanoic acid methyl ester | yes | no | Differential markers only |
| linalool | yes | yes | Intersection (core putative markers) |
| 1-hexanol | yes | no | Differential markers only |
| 3-methyl-1-butanol | yes | no | Differential markers only |
| 4-methyl-2-pentanol | no | yes | Aroma candidates only |
| 2-Octanone | no | yes | Aroma candidates only |
| 2,3-butanedione | no | yes | Aroma candidates only |
| 3-heptanone | no | yes | Aroma candidates only |
| 2-pentanone | no | yes | Aroma candidates only |
| 1-hydroxy-2-propanone | no | yes | Aroma candidates only |
| 1-penten-3-one | no | yes | Aroma candidates only |
| ethyl acetate | no | yes | Aroma candidates only |
| butyl acetate | no | yes | Aroma candidates only |
| ethyl butanoate | no | yes | Aroma candidates only |
| 2-methylbutanoic acid ethyl ester | no | yes | Aroma candidates only |
| nonanal | no | yes | Aroma candidates only |
| Benzaldehyde | no | yes | Aroma candidates only |
| heptanal | no | yes | Aroma candidates only |
| 2-methylpropanal | no | yes | Aroma candidates only |
| pentanal | no | yes | Aroma candidates only |
| propanal | no | yes | Aroma candidates only |
| butanal | no | yes | Aroma candidates only |
| (E)-2-hexenal | no | yes | Aroma candidates only |
| styrene | no | yes | Aroma candidates only |
| alpha-pinene | no | yes | Aroma candidates only |
| dimethyl disulfide | no | yes | Aroma candidates only |
| allyl sulfide | no | yes | Aroma candidates only |
| dimethyl sulfide | no | yes | Aroma candidates only |
| (E)-2-nonenal | no | yes | Aroma candidates only |
| decanal | no | yes | Aroma candidates only |
| damascenone | no | yes | Aroma candidates only |
| 3-methyl-phenol | no | yes | Aroma candidates only |
| Anisole | no | yes | Aroma candidates only |
