# Supplementary material for: Analysis of characteristic flavor compounds in coffee peels subjected to different thermal processing treatments based on GC–IMS and HS-SPME–GC–MS combined with chemical pattern recognition
Source: Food Chem X. 2026 Aug 10;38:104256. doi: 10.1016/j.fochx.2026.104256 (PMC13524620; doi:10.1016/j.fochx.2026.104256)
Supplement: Supplementary file 2 [file mmc2.docx]

Table S2. Table of traceable odour-threshold source

| VOCs | Odor threshold（mg/kg) | Aroma properties | ROAV/% | | | Reported source | Original matrix |
| --- | --- | --- | --- | --- | --- | --- | --- |
|  |  |  | SG | HAD | MD |  |  |
| 1-hexanol | 0.0056 | Fruity | 3.23 | 1.74 | 1.44 | van Gemert (2003) | Water (as reported in previous manuscript) |
| 3-methyl-1-butanol | 0.004 | Fruity | 4.16 | 0.51 | 0.48 |  |  |
| 4-methyl-2-pentanol | 2.5 | Sour/Fermented | 0.00 | 0.00 | 1.09 |  |  |
| 2-furanmethanethiol | 0.00008 | Roasted | 100.00 | 100.00 | 100.00 |  |  |
| 2-Octanone | 0.0502 | Other | 0.79 | 0.27 | 0.17 |  |  |
| 2,3-butanedione | 0.00774 | Other | 3.04 | 0.29 | 0.37 |  |  |
| 3-heptanone | 0.08 | Fruity | 0.22 | 0.04 | 0.03 |  |  |
| 2-pentanone | 1.38 | Fruity | 0.01 | 0.00 | 0.97 |  |  |
| 1-hydroxy-2-propanone | 10 | Sweet | 0.01 | 0.83 | 0.00 |  |  |
| 1-penten-3-one | 0.023 | Spices | 1.03 | 0.18 | 0.16 |  |  |
| ethyl acetate | 0.005 | Fruity | 2.82 | 0.24 | 0.26 |  |  |
| butyl acetate | 0.058 | Fruity | 0.08 | 0.19 | 0.08 |  |  |
| ethyl butanoate | 0.0009 | Fruity | 8.27 | 7.26 | 4.74 |  |  |
| ethyl heptanoate | 0.0019 | Fruity | 1.00 | 0.61 | 0.22 |  |  |
| 2-methylbutanoic acid ethyl ester | 0.000063 | Fruity | 98.62 | 15.62 | 11.15 |  |  |
| nonanal | 0.0011 | Floral | 16.68 | 6.93 | 6.21 |  |  |
| (E)-2-decenal | 0.0003 | Other | 47.93 | 12.04 | 13.42 |  |  |
| 3-methylbutanal | 0.0011 | Nutty/Cocoa | 56.35 | 15.35 | 14.27 |  |  |
| Benzaldehyde | 0.32 | Nutty/Cocoa | 1.31 | 0.15 | 0.13 |  |  |
| heptanal | 0.0028 | Green/Vegetative | 7.19 | 0.62 | 0.75 |  |  |
| hexanal | 0.005 | Green/Vegetative | 4.33 | 0.58 | 0.74 |  |  |
| 2-methylpropanal | 0.0015 | Green/Vegetative | 21.25 | 9.05 | 6.53 |  |  |
| pentanal | 0.012 | Sour/Fermented | 0.89 | 0.18 | 0.15 |  |  |
| propanal | 0.0151 | Sour/Fermented | 0.59 | 0.08 | 0.09 |  |  |
| butanal | 0.000182 | Nutty/Cocoa | 55.49 | 12.60 | 11.09 |  |  |
| (E)-2-hexenal | 0.0887 | Green/Vegetative | 0.55 | 0.03 | 0.04 |  |  |
| styrene | 0.065 | Other | 0.06 | 0.16 | 0.06 |  |  |
| alpha-pinene | 0.014 | Other | 2.88 | 3.99 | 3.26 |  |  |
| dimethyl disulfide | 0.0011 | Green/Vegetative | 18.53 | 5.12 | 4.43 |  |  |
| allyl sulfide | 0.1 | Other | 0.23 | 0.06 | 0.05 |  |  |
| dimethyl sulfide | 0.00012 | Green/Vegetative | 36.71 | 18.19 | 11.14 |  |  |
| (E)-2-nonenal | 0.00019 | Green/Vegetative | 0.00 | 3.07 | 2.52 |  |  |
| decanal | 0.003 | Fruity | 0.00 | 0.46 | 0.36 |  |  |
| damascenone | 0.000126 | Floral | 0.21 | 8.29 | 4.87 |  |  |
| β-Ionone | 0.000007 | Floral | 100.00 | - | - |  |  |
| Benzyl alcohol | 0.0001 | Floral | 14.21 | 100.00 | 100.00 |  |  |
| Linalool | 0.00022 | Floral | 3.45 | 26.90 | 23.88 |  |  |
| 3-methyl-phenol | 0.031 | Other | 0.01 | 0.11 | - |  |  |
| Anisole | 0.1775 | Other | 0.01 | 0.15 | 0.12 |  |  |
