# Supplementary material for: Analysis of characteristic flavor compounds in coffee peels subjected to different thermal processing treatments based on GC–IMS and HS-SPME–GC–MS combined with chemical pattern recognition
Source: Food Chem X. 2026 Aug 10;38:104256. doi: 10.1016/j.fochx.2026.104256 (PMC13524620; doi:10.1016/j.fochx.2026.104256)
Supplement: Supplementary file 3 [file mmc3.docx]

Tab.1 GC-IMS analysis results of the contents and types of volatile substances in coffee peels

| Classification | VOCs | CAS | Molecular formula | Molecular  Weight  (g/mol) | Retention Index | Retention  Time/s | Drift Time/ms | Relative amount/% | | |
| --- | --- | --- | --- | --- | --- | --- | --- | --- | --- | --- |
|  |  |  |  |  |  |  |  | SG | HX | WB |
| Alcohols /13 species | 1-Hexanol | C111273 | C6H14O | 102.2 | 1341.1 | 841.061 | 1.3118 | 0.871±0.040^b^ | 1.774±0.556^a^ | 1.910±0.295^a^ |
|  | 2-furanmethanol | C98000 | C5H6O2 | 98.1 | 1663.7 | 1763.062 | 1.35863 | 3.087±0.456^ab^ | 3.362±0.382^a^ | 2.698±0.170^b^ |
|  | 3-methyl-1-Butanol | C123513 | C5H12O | 88.1 | 1215.3 | 632.224 | 1.24566 | 0.801±0.020^a^ | 0.368±0.019^c^ | 0.455±0.014^b^ |
|  | 1-Propanol | C71238 | C3H8O | 60.1 | 1030.4 | 355.941 | 1.10977 | 0.995±0.048^a^ | 0.566±0.066^b^ | 0.513±0.010^b^ |
|  | 2-Methyl-1-propanol_M | C78831 | C4H10O | 74.1 | 1091.8 | 426.386 | 1.17559 | 0.852±0.018^a^ | 0.327±0.009^c^ | 0.386±0.007^b^ |
|  | 2-methyl-1-propanol_D | C78831 | C4H10O | 74.1 | 1092.3 | 427.143 | 1.37279 | 0.273±0.011^a^ | 0.158±0.006^c^ | 0.216±0.005^b^ |
|  | ethanol | C64175 | C2H6O | 46.1 | 936.3 | 273.969 | 1.14563 | 6.231±0.215^a^ | 1.987±0.100^c^ | 2.444±0.024^b^ |
|  | 4-methyl-2-pentanol | C108112 | C6H14O | 102.2 | 1185.2 | 590.863 | 1.28018 | 0.339±0.018^a^ | 0.184±0.014^b^ | 0.191±0.005^b^ |
|  | 2-methyl isoborneol | C2371428 | C11H20O | 168.3 | 1598.3 | 1517.324 | 1.26609 | 0.018±0.008^c^ | 0.259±0.028^a^ | 0.194±0.013^b^ |
|  | 3-methyl-2-butanol | C598754 | C5H12O | 88.1 | 1094 | 429.764 | 1.44032 | 0.186±0.010^c^ | 0.224±0.010^b^ | 0.316±0.005^a^ |
|  | 1-Penten-3-ol | C616251 | C5H10O | 86.1 | 1157.4 | 538.057 | 0.94405 | 1.092±0.020^a^ | 0.776±0.070^c^ | 0.889±0.020^b^ |
|  | 3-Methyl-3-buten-1-ol | C763326 | C5H10O | 86.1 | 1253.9 | 689.736 | 1.43539 | 0.086±0.003^c^ | 0.270±0.026^a^ | 0.221±0.012^b^ |
|  | 2-furanmethanethiol | C98022 | C5H6OS | 114.2 | 1414.3 | 994.743 | 1.10539 | 0.385±0.022^c^ | 1.455±0.025^b^ | 1.891±0.020^a^ |
| Ketones/15 species | 2-Octanone | C111137 | C8H16O | 128.2 | 1292.8 | 752.853 | 1.32882 | 1.909±0.066^c^ | 2.436±0.088^a^ | 2.061±0.008^b^ |
|  | 1-(2-furanyl)-ethanone_M | C1192627 | C6H6O2 | 110.1 | 1520.2 | 1268.432 | 1.1252 | 0.549±0.028^c^ | 2.918±0.053^a^ | 2.301±0.033^b^ |
|  | 1-(2-furanyl)-ethanone_D | C1192627 | C6H6O2 | 110.1 | 1520 | 1267.862 | 1.44146 | 0.245±0.015^c^ | 0.515±0.038^a^ | 0.319±0.005^b^ |
|  | 2-Butanone | C78933 | C4H8O | 72.1 | 913.4 | 257.568 | 1.24419 | 0.724±0.021^c^ | 1.675±0.023^b^ | 1.871±0.018^a^ |
|  | 2,3-Butanedione | C431038 | C4H6O2 | 86.1 | 955.1 | 288.13 | 1.1805 | 1.134±0.054^a^ | 0.412±0.009^c^ | 0.685±0.009^b^ |
|  | 3-heptanone | C106354 | C7H14O | 114.2 | 1160.4 | 543.905 | 1.21671 | 0.832±0.044^a^ | 0.589±0.007^b^ | 0.560±0.008^b^ |
|  | 2-pentanone | C107879 | C5H10O | 86.1 | 983.7 | 311.178 | 1.1226 | 0.883±0.028^a^ | 0.848±0.018^b^ | 0.833±0.017^b^ |
|  | 1-hydroxy-2-propanone_M | C116096 | C3H6O2 | 74.1 | 1309.3 | 781.839 | 1.07327 | 2.818±0.046^b^ | 2.862±0.196^b^ | 3.164±0.079^a^ |
|  | 1-hydroxy-2-propanone_D | C116096 | C3H6O2 | 74.1 | 1309 | 781.268 | 1.23014 | 0.785±0.044^b^ | 4.934±0.357^a^ | 4.835±0.133^a^ |
|  | Cyclopentanone | C120923 | C5H8O | 84.1 | 1185.6 | 591.4 | 1.10774 | 0.111±0.005^c^ | 0.178±0.008^b^ | 0.208±0.004^a^ |
|  | 4-methyl-3-penten-2-one | C141797 | C6H10O | 98.1 | 1126.5 | 482.209 | 1.12434 | 1.861±0.043^a^ | 0.298±0.011^b^ | 0.298±0.010^b^ |
|  | 1-penten-3-one | C1629589 | C5H8O | 84.1 | 1025.8 | 351.26 | 1.30933 | 1.144±0.060^a^ | 0.755±0.019^c^ | 0.845±0.008^b^ |
|  | alpha-Thujone | C546805 | C10H16O | 152.2 | 1414.6 | 995.418 | 1.37139 | 0.257±0.019^c^ | 0.328±0.017^b^ | 0.478±0.013^a^ |
|  | 5-methyl-2(3H)-Furanone | C591128 | C5H6O2 | 98.1 | 1434.4 | 1041.622 | 1.12641 | 0.165±0.027^c^ | 0.383±0.013^b^ | 0.485±0.007^a^ |
|  | 3-pentanone | C96220 | C5H10O | 86.1 | 984 | 311.455 | 1.35248 | 0.162±0.008^c^ | 0.267±0.014^b^ | 0.309±0.004^a^ |
| Esters/12 species | gamma-Butyrolactone_M | C96480 | C4H6O2 | 86.1 | 1668.4 | 1782.041 | 1.08775 | 9.457±0.146^a^ | 8.542±0.123^b^ | 6.841±0.042^c^ |
|  | gamma-Butyrolactone_D | C96480 | C4H6O2 | 86.1 | 1667.7 | 1779.121 | 1.30626 | 1.736±0.110^b^ | 2.873±0.258^a^ | 1.814±0.062^b^ |
|  | Ethyl acetate | C141786 | C4H8O2 | 88.1 | 897 | 246.445 | 1.3363 | 0.678±0.055^a^ | 0.222±0.008^c^ | 0.310±0.008^b^ |
|  | Butyl acetate | C123864 | C6H12O2 | 116.2 | 1063.5 | 391.76 | 1.24184 | 0.236±0.019^c^ | 1.997±0.162^a^ | 1.075±0.074^b^ |
|  | propyl acetate | C109604 | C5H10O2 | 102.1 | 982.3 | 310.014 | 1.16135 | 3.220±0.075^a^ | 1.877±0.023^c^ | 2.877±0.024^b^ |
|  | Ethyl butanoate | C105544 | C6H12O2 | 116.2 | 1047.9 | 374.478 | 1.20318 | 0.358±0.021^c^ | 1.189±0.021^a^ | 1.009±0.007^b^ |
|  | Ethyl heptanoate | C106309 | C9H18O2 | 158.2 | 1328.7 | 817.297 | 1.41037 | 0.091±0.007^b^ | 0.211±0.037^a^ | 0.098±0.005^b^ |
|  | Ethyl formate | C109944 | C3H6O2 | 74.1 | 834.6 | 208.366 | 1.07603 | 2.716±0.093^a^ | 1.310±0.049^c^ | 1.513±0.014^b^ |
|  | Methyl propanoate | C554121 | C4H8O2 | 88.1 | 913.8 | 257.882 | 1.10339 | 2.079±0.062^a^ | 1.778±0.038^c^ | 1.875±0.012^b^ |
|  | butyl propanoate | C590012 | C7H14O2 | 130.2 | 1140.1 | 506.041 | 1.28634 | 0.120±0.010^b^ | 0.108±0.005^c^ | 0.168±0.005^a^ |
|  | ethyl(E)-2-butenoate | C623701 | C6H10O2 | 114.1 | 1161 | 545.054 | 1.18177 | 0.086±0.006^c^ | 0.110±0.004^a^ | 0.092±0.002^b^ |
|  | 2-methylbutanoic acid ethyl ester | C7452791 | C7H14O2 | 130.2 | 1051.6 | 378.498 | 1.2387 | 0.299±0.034^a^ | 0.179±0.016^b^ | 0.166±0.011^b^ |
| Aldehydes/14 species | Nonanal | C124196 | C9H18O | 142.2 | 1398.3 | 958.935 | 1.48283 | 0.883±0.024^a^ | 0.333±0.013^c^ | 0.439±0.009^b^ |
|  | (E)-2-decenal | C3913813 | C10H18O | 154.3 | 1616.4 | 1581.444 | 1.48283 | 0.692±0.016^b^ | 0.657±0.037^b^ | 0.952±0.028^a^ |
|  | Furfural | C98011 | C5H4O2 | 96.1 | 1480.5 | 1157.91 | 1.33825 | 1.725±0.060^c^ | 15.398±0.163^a^ | 14.873±0.068^b^ |
|  | 3-Methylbutanal | C590863 | C5H10O | 86.1 | 926.1 | 266.549 | 1.40907 | 2.983±0.086^c^ | 3.070±0.040^b^ | 3.710±0.041^a^ |
|  | Benzaldehyde | C100527 | C7H6O | 106.1 | 1482.9 | 1164.285 | 1.15453 | 20.213±0.815^a^ | 8.927±0.274^c^ | 9.649±0.044^b^ |
|  | Heptanal_M | C111717 | C7H14O | 114.2 | 1185.5 | 591.303 | 1.34099 | 0.726±0.024^a^ | 0.218±0.013^c^ | 0.369±0.018^b^ |
|  | Heptanal_D | C111717 | C7H14O | 114.2 | 1185.9 | 591.839 | 1.68222 | 0.243±0.010^a^ | 0.100±0.007^c^ | 0.126±0.005^b^ |
|  | Hexanal | C66251 | C6H12O | 100.2 | 1088.1 | 420.857 | 1.56019 | 1.042±0.031^a^ | 0.528±0.007^c^ | 0.869±0.010^b^ |
|  | 2-Methylpropanal | C78842 | C4H8O | 72.1 | 817.5 | 199.003 | 1.08782 | 1.534±0.040^c^ | 2.469±0.048^a^ | 2.314±0.020^b^ |
|  | pentanal | C110623 | C5H10O | 86.1 | 985.8 | 312.919 | 1.42329 | 0.515±0.014^a^ | 0.391±0.018^c^ | 0.426±0.005^b^ |
|  | Propanal | C123386 | C3H6O | 58.1 | 812.7 | 196.492 | 1.03955 | 0.426±0.026^a^ | 0.222±0.011^c^ | 0.307±0.004^b^ |
|  | Butanal | C123728 | C4H8O | 72.1 | 892.4 | 243.439 | 1.13714 | 0.486±0.016^a^ | 0.417±0.002^b^ | 0.477±0.006^a^ |
|  | (E)-2-Hexenal_M | C6728263 | C6H10O | 98.1 | 1230 | 653.523 | 1.18301 | 1.776±0.047^a^ | 0.408±0.016^c^ | 0.724±0.019^b^ |
|  | (E)-2-Hexenal_D | C6728263 | C6H10O | 98.1 | 1229.9 | 653.449 | 1.52326 | 0.591±0.036^a^ | 0.095±0.004^c^ | 0.188±0.004^b^ |
| Hydrocarbons/5 species | ethylbenzene | C100414 | C8H10 | 106.2 | 1129.2 | 486.778 | 1.07637 | 1.352±0.050^c^ | 1.510±0.026^b^ | 1.680±0.022^a^ |
|  | styrene | C100425 | C8H8 | 104.2 | 1275.9 | 724.687 | 1.42003 | 0.195±0.011^c^ | 1.883±0.128^a^ | 0.980±0.027^b^ |
|  | Propylbenzene | C103651 | C9H12 | 120.2 | 1180.7 | 584.384 | 1.24409 | 0.148±0.005^a^ | 0.127±0.030^a^ | 0.073±0.009^b^ |
|  | alpha-Pinene | C80568 | C10H16 | 136.2 | 1021.4 | 346.856 | 1.21325 | 1.703±0.069^a^ | 1.070±0.038^c^ | 1.162±0.014^b^ |
|  | gamma-Terpinene | C99854 | C10H16 | 136.2 | 1254.6 | 690.692 | 1.21747 | 0.182±0.008^c^ | 0.961±0.054^a^ | 0.897±0.022^b^ |
| Ethers/4 species | dimethyl disulfide | C624920 | C2H6S2 | 94.2 | 1037.6 | 363.492 | 1.14844 | 0.981±0.054^b^ | 1.024±0.036^b^ | 1.153±0.012^a^ |
|  | 1,2-Dimethoxyethane | C110714 | C4H10O2 | 90.1 | 932.2 | 270.96 | 1.30321 | 0.739±0.023^b^ | 0.686±0.008^c^ | 0.812±0.010^a^ |
|  | Allyl sulfide | C592881 | C6H10S | 114.2 | 1136.1 | 498.958 | 1.12582 | 1.089±0.060^b^ | 1.153±0.023^a^ | 1.092±0.014^b^ |
|  | Dimethyl sulfide | C75183 | C2H6S | 62.1 | 743.8 | 163.224 | 0.96286 | 0.212±0.022^c^ | 0.397±0.029^a^ | 0.316±0.014^b^ |
| Pyrazines/2 species | 2-Methylpyrazine | C109080 | C5H6N2 | 94.1 | 1274.8 | 722.864 | 1.0851 | 0.886±0.041^c^ | 1.738±0.089^b^ | 2.004±0.032^a^ |
|  | 2,3-dimethyl-5-ethylpyrazine | C15707343 | C8H12N2 | 136.2 | 1481.3 | 1160.015 | 1.2262 | 1.630±0.088^b^ | 3.144±0.079^a^ | 3.177±0.030^a^ |
| Furans/1species | 2-ethyl furan | C3208160 | C6H8O | 96.1 | 955.5 | 288.488 | 1.30423 | 0.046±0.002^c^ | 0.176±0.005^b^ | 0.200±0.004^a^ |
| Acids/1 species | acetic acid | C64197 | C2H4O2 | 60.1 | 1475.9 | 1145.739 | 1.05924 | 6.772±0.379^a^ | 1.101±0.052^c^ | 1.387±0.022^b^ |
| Heterocycles/1 species | 1,4-dioxane | C123911 | C4H8O2 | 88.1 | 1088.5 | 421.379 | 421.379 | 0.230±0.010^b^ | 0.290±0.007^a^ | 0.230±0.011^b^ |

note a: "-" indicates that the aroma description of the organism could not be found;

note b: Different lowercase letters denote significant differences in the comparison of the relative content of the same VOCs in different samples, respectively (P < 0.05).
