# Supplementary material for: Analysis of characteristic flavor compounds in coffee peels subjected to different thermal processing treatments based on GC–IMS and HS-SPME–GC–MS combined with chemical pattern recognition
Source: Food Chem X. 2026 Aug 10;38:104256. doi: 10.1016/j.fochx.2026.104256 (PMC13524620; doi:10.1016/j.fochx.2026.104256)
Supplement: Supplementary file 4 [file mmc4.docx]

Tab.2 HS-SPME-GC-MS analysis results of the contents and types of volatile substances in coffe peels

| Classification | VOCs | CAS | Retention Time/min | Relative amount/% | | |
| --- | --- | --- | --- | --- | --- | --- |
|  |  |  |  | SG | HX | WB |
| Aldehydes/9 species | Furfural | C98011 | 5.421 | 5.278±0.195^c^ | 11.677±0.163^b^ | 13.290±0.295^a^ |
|  | 5-methyl-2-Furancarboxaldehyde | C620020 | 8.674 | - | 6.335±0.127^b^ | 9.020±0.234^a^ |
|  | Nonanal | C124196 | 12.833 | 4.098±0.074^a^ | 1.985±0.040^c^ | 2.283±0.114^b^ |
|  | (+)-Citronellal | C[2385775](https://www.chemsrc.com/baike/684521.html) | 14.159 | 0.842±0.045^a^ | - | 0.423±0.040^b^ |
|  | (E)-2-Nonenal | C18829566 | 14.384 | - | 0.200±0.024^a^ | 0.220±0.021^a^ |
|  | Decanal | C112312 | 15.669 | 1.072±0.070^a^ | 0.473±0.023^b^ | 0.492±0.085^b^ |
|  | 5-Hydroxymethylfurfural | C67470 | 16.455 | - | 0.517±0.046^a^ | 0.228±0.049^b^ |
|  | 2-Isopropyl-5-methylhex-4-enal | C58191814 | 16.778 | - | - | 0.327±0.036^a^ |
|  | Citral | C5392405 | 17.293 | 1.255±0.088^a^ | 0.910±0.054^b^ | 0.723±0.052^c^ |
| Ketones/14 species | 4-Cyclopentene-1,3-dione | C[930609](https://www.chemsrc.com/baike/1195557.html) | 6.452 | - | 1.253±0.029^b^ | 1.752±0.043^a^ |
|  | 1-(2-furanyl)-Ethanone | C1192627 | 7.272 | 0.613±0.050^c^ | 2.352±0.099^b^ | 2.897±0.140^a^ |
|  | 6-methyl-5-Hepten-2-one | C[110930](https://www.chemsrc.com/baike/509694.html) | 9.225 | 3.872±0.040^a^ | 1.560±0.041^c^ | 1.770±0.123^b^ |
|  | 1,2-Cyclooctanedione | C3008375 | 11.370 | - | - | 0.233±0.015^a^ |
|  | 1-(1H-pyrrol-2-yl)-Ethanone | C1072839 | 11.571 | 0.703±0.100^c^ | 3.572±0.075^b^ | 3.895±0.091^a^ |
|  | 2,5-Dimethylfuran-3,4(2H,5H)-dione | C68755497 | 11.799 | 0.275±0.019^c^ | 6.958±0.215^a^ | 0.598±0.066^b^ |
|  | 1-(2-pyridinyl)-1-Propanone | C3238559 | 13.734 | - | 0.260±0.031^a^ | - |
|  | 2,3-dihydro-3,5-dihydroxy-6-methyl-4H-Pyran-4-one | C28564832 | 13.951 | 1.683±0.095^c^ | 27.062±0.438^a^ | 19.543±1.622^b^ |
|  | 3,5-dihydroxy-2-methyl-4H-Pyran-4-one | C1073967 | 15.362 | 0.428±0.041^c^ | 0.748±0.049^a^ | 0.657±0.034^b^ |
|  | Damascenone | C23696857 | 20.571 | 0.223±0.022^c^ | 0.358±0.010^a^ | 0.282±0.008^b^ |
|  | alpha.-Ionone | C[127413](https://www.chemsrc.com/baike/509667.html) | 21.647 | - | 0.027±0.005^a^ | 0.028±0.004^a^ |
|  | Geranylacetone | C689678 | 22.202 | 0.113±0.037^a^ | 0.057±0.005^b^ | 0.057±0.005^b^ |
|  | beta.-Ionone | C[14901076](https://www.chemsrc.com/baike/342032.html) | 23.115 | 5.808±0.069^a^ | - | - |
|  | 6,10,14-trimethyl-2-Pentadecanone | C502692 | 30.739 | 0.048±0.012^a^ | 0.025±0.005^b^ | 0.027±0.005^b^ |
| Alcohols /8 species | 2-Furanmethanol | C98000 | 5.814 | - | 4.880±0.050^b^ | 5.848±0.088^a^ |
|  | 5-methyl-2-Furanmethanol | C3857258 | 8.426 | - | 1.923±0.061^b^ | 2.028±0.033^a^ |
|  | 3-Ethyl-4-methylpentan-1-ol | C38514135 | 10.305 | 0.205±0.012^b^ | 0.267±0.010^b^ | 0.815±0.128^a^ |
|  | Benzyl alcohol | C100516 | 10.783 | 11.790±0.142^a^ | 3.427±0.090^c^ | 4.592±0.121^b^ |
|  | trans-Linalool oxide(furanoid) | C34995772 | 12.366 | 3.275±0.092^a^ | 1.492±0.026^c^ | 1.615±0.078^b^ |
|  | Linalool | C78706 | 12.610 | 6.302±0.090^a^ | 2.028±0.048^c^ | 2.412±0.090^b^ |
|  | Phenylethyl Alcohol | C60128 | 13.053 | 6.760±0.116^a^ | - | - |
|  | (3R,6S)-2,2,6-Trimethyl-6-vinyltetrahydro-2H-pyran-3-ol | C14049117 | 14.784 | 2.787±0.107^a^ | 1.675±0.030^c^ | 1.955±0.038^b^ |
| Esters/11 species | Furfuryl acetate | C[623176](https://www.chemsrc.com/baike/832508.html) | 9.735 | - | - | 0.745±0.086^a^ |
|  | Octanoic acid methyl ester | C111115 | 13.301 | 0.958±0.076^a^ | 0.265±0.021^b^ | 0.183±0.036^c^ |
|  | Benzyl acetate | C[140114](https://www.chemsrc.com/baike/509664.html) | 14.515 | 5.178±0.043^a^ | - | - |
|  | Nonanoic acid methyl ester | C1731846 | 16.153 | 0.525±0.046^a^ | 0.300±0.017^b^ | 0.273±0.008^b^ |
|  | Nonanoic acid ethyl ester | C[123295](https://www.chemsrc.com/baike/237412.html) | 18.115 | 0.083±0.021^a^ | - | 0.027±0.005^b^ |
|  | 3-hydroxy-2,2,4-trimethylpentyl isobutyrate | C[25265774](https://www.chemsrc.com/baike/1085289.html) | 20.240 | 0.295±0.036^a^ | 0.088±0.028^b^ | 0.110±0.036^b^ |
|  | Methyl tetradecanoate | C124107 | 28.794 | 0.130±0.021^a^ | 0.078±0.004^b^ | 0.082±0.010^b^ |
|  | 2-Ethylhexyl salicylate | C[118605](https://www.chemsrc.com/baike/442736.html) | 30.255 | - | 0.020±0.009^a^ | 0.023±0.005^a^ |
|  | Homosalate | C118569 | 31.452 | 0.050±0.009^a^ | 0.020±0.000^b^ | 0.020±0.000^b^ |
|  | Hexadecanoic acid methyl ester | C112390 | 31.823 | 1.077±0.038^a^ | 0.512±0.035^c^ | 0.698±0.051^b^ |
|  | Hexadecanoic acid ethyl ester | C628977 | 32.638 | 0.073±0.010^a^ | 0.032±0.008^b^ | 0.028±0.008^b^ |
| Acids/4 species | Octanoic acid | C124072 | 13.527 | - | - | 0.243±0.016^a^ |
|  | Nonanoic acid | C112050 | 17.590 | - | - | 0.692±0.096^a^ |
|  | Decanoic acid | C[334485](https://www.chemsrc.com/baike/832315.html) | 20.059 | - | - | 0.330±0.059^a^ |
|  | Dodecanoic acid | C143077 | 24.880 | - | 0.037±0.008^a^ | - |
| Olefins/4 species | Styrene | C100425 | 6.820 | - | - | 1.600±0.041^a^ |
|  | (+)-alpha-Pinene | C[7785708](https://www.chemsrc.com/baike/1042148.html) | 7.850 | 6.347±0.183^a^ | 2.690±0.090^c^ | 2.943±0.150^b^ |
|  | β-pinene | C[18172673](https://www.chemsrc.com/baike/88283.html) | 9.415 | 8.553±0.260^a^ | 3.060±0.092^b^ | 2.782±0.104^c^ |
|  | beta.-Ocimene | C13877913 | 11.073 | 0.477±0.061^a^ | 0.247±0.038^c^ | 0.375±0.037^b^ |
| Phenols/3 species | 3-methyl-Phenol | C108394 | 12.062 | 1.635±0.069^a^ | 1.203±0.016^b^ | - |
|  | 5-ethenyl-2-methoxy-Phenol | C621589 | 18.730 | - | 0.152±0.004^b^ | 0.175±0.008^a^ |
|  | 2,4-Di-tert-butylphenol | C96764 | 23.726 | 0.180±0.023^a^ | 0.067±0.005^b^ | 0.080±0.017^b^ |
| Ethers/1 species | Anisole | C100663 | 7.419 | 16.797±0.130^a^ | 8.827±0.159^c^ | 10.005±0.147^b^ |
| Others/1 species | Caffeine | C[58082](https://www.chemsrc.com/baike/895838.html) | 31.057 | 0.210±0.037^c^ | 0.292±0.033^b^ | 0.450±0.029^a^ |
| Pyrrole/1 species | 1-(2-furanylmethyl)-1H-Pyrrole | C1438944 | 15.077 | - | 0.092±0.030^b^ | 0.125±0.029^a^ |

note a: "-" indicates that the aroma description of the organism could not be found;

note b: Different lowercase letters denote significant differences in the comparison of the relative content of the same VOCs in different samples, respectively (P < 0.05).
